# New chalcone compound exhibits microrna-mediated anticancer properties in glioblastoma

**DOI:** 10.1371/journal.pone.0330624

**Published:** 2025-09-26

**Authors:** Denis Mustafov, Shoib S. Siddiqui, Shagufta, Irshad Ahmad, Joanne Charlton, Jim Gkantalis, Patricia Rodriguez, Maria Braoudaki

**Affiliations:** 1 School of Life and Medical Sciences, University of Hertfordshire, Hatfield, United Kingdom; 2 College of Health, Medicine and Life Sciences, Brunel University London, Uxbridge, United Kingdom; 3 Department of Biotechnology, School of Arts and Sciences, American University of Ras Al Khaimah, Ras Al Khaimah, United Arab Emirates; 4 Facultad de Ciencias Experimentales, Universidad Francisco de Vitoria, Carretera Pozuelo a Majadahonda, Km, Pozuelo de Alarcón, Madrid, España; IIIT Kurnool: Indian Institute of Information Technology Design and Manufacturing Kurnool, INDIA

## Abstract

**Background:**

Therapy-resistant glioblastoma constitutes the most lethal adult brain malignancy, bearing extremely poor patients’ prognosis. Combinational therapies are accompanied by severe side effects and significant financial burden, highlighting the urgent need for alternative, low toxicity treatments. This study sought to investigate the therapeutic efficacy of a new synthetic chalcone molecule, SHG-44, and its cellular mechanistic actions in U87MG and U251MG glioblastoma cell line models.

**Methods:**

The effect of SHG-44 on glioblastoma cell viability, clonogenic survival, and cell migration was assessed using *in vitro* functional assays. Cellular reactive oxygen species levels were quantified to investigate oxidative stress induced by SHG-44. The induction of apoptosis by SHG-44 was examined through nuclear fragmentation analysis and acridine orange/ethidium bromide staining. Small RNA-sequencing was performed to explore the regulatory effects of SHG44 on microRNA expression. The pharmacokinetic and pharmacodynamic properties of SHG-44 were evaluated through *in-silico* analysis.

**Results:**

Cell viability assays demonstrated that SHG-44 significantly reduced cell viability in glioblastoma cells, with half-maximal inhibitory concentration of 70.39ΜΜ and 64.19ΜΜ in U87MG and U251MG cells, respectively. In line with these findings, a colony formation assay revealed a significant impairment of glioblastoma cells’ clonogenic survival. SHG-44 effects on cell migration demonstrated a substantial abrogation of cell migration at 100ΜΜ SHG-44 within both glioblastoma cell lines. Cellular reactive oxygen species quantification showed a significant increase of ROS at 40ΜΜ SHG-44 concentrations within U87MG cells and a non-significant elevation of ROS within U251MG cells. Nuclear fragmentation and acridine orange/ethidium bromide staining demonstrated that SHG-44 mediated cytotoxicity in U87MG and U251MG through apoptosis induction. Small RNA-sequencing analysis demonstrated that SHG-44 could regulate various cellular pathways through microRNA expression modulation in glioblastoma cell models. Further *in-silico* analysis of the pharmacokinetics and pharmacodynamics properties of SHG-44 demonstrated that the compound possessed sufficient absorption, distribution, potential to pass through the blood brain barrier, and low oral toxicity characteristics.

**Conclusion:**

Taken together these findings suggested the potential anti-cancer properties of the newly synthesized chalcone compound, SHG-44 and its dynamic implications in glioblastoma therapeutic management.

## Introduction

Glioblastoma (GB) is the most prevalent and aggressive form of primary brain neoplasm in adults. The fifth edition of the World Health Organization (WHO) classification of tumours of the central nervous system (CNS) published in 2021 categorizes GB as a grade 4 brain malignancy [1]. The aggressiveness of GB is attributed to its rapid proliferation, diffuse infiltration, and intra-tumoral heterogeneity, resulting in a poor prognosis, with a shallow 5-year survival rate of only 5% and a median survival of approximately 15 months [2].

Despite advancements in intraoperative guiding imaging technologies that have revolutionized GB surgical resection, achieving gross total resection remains a challenge due to the infiltrative nature of the tumour and indistinct margins within adjacent cortical areas [3,4]. Consequently, the current gold-standard post-operative treatment for GB combines locoregional radiotherapy with systemic temozolomide (TMZ) chemotherapy [5]. However, GB is well-known for its resistance to radiotherapy and other DNA-damaging agents, partly attributable to the tumour microenvironment’s heterogeneity and the intrinsic proficiency of GB cells in repairing DNA double-strand breaks [6]. The complexity of GB highlights the critical need for the development of innovative therapeutic approaches.

Chalcones, a class of unsaturated ketones belonging to the diverse bioactive flavonoid family of polyphenols, exhibit promising anti-cancer properties with potential medical applications [7]. Due to their simple chemistry, ease of synthesis, and modifiable structure, chalcones are particularly promising in cancer treatment. Their molecular targets correlating with their anticancer among others, include ATP binding cassette subfamily G (ABCG2), tubulin, activated nuclear B cell growth (NF-κB), vascular endothelial growth factor (VEGF), tyrosine kinase receptor (EGFR), and mesenchymal-epithelial transition factor (MET) [8]. A recent publication investigating the role of a new chalcone derivative, HCTMPPK, for the treatment of lung cancer demonstrated its tumour suppressive properties both *in vitro* and *in vivo* [9]. In addition, chalcone derivative (1) has shown promising anti-cancer effects upon GB cell models via exhibiting cytotoxic, antiproliferative, and anti-invasion activities [10]. The selected chalcone derivative induced apoptosis in GB cells by causing cell cycle arrest at the G2/M checkpoint.

Furthermore, a recent report has demonstrated that a chalcone derivative from hops named Xanthohumol (XN), kills glioblastoma cells [11]. The study also revealed that XN triggers cell death and targets miR-204-3p, an established tumour suppressor in GB that increases with XN treatment. Nevertheless, research on the therapeutic potential of chalcones in GB remains limited.

To date, there is a vast amount of evidence that altering the expression profiles of deregulated microRNAs (miRNAs) within GB rescues the phenotype of cancer cells and seizes tumour growth [12]. However, there is limited evidence on how chalcone derivatives regulate the miRNome within GB cells. MiRNAs are small RNA transcripts with a size of about 18–22 nucleotides that have emerged as a promising therapeutic strategy for GB due to their ability to regulate a variety of genes involved in tumorigenesis [13]. The dysregulation of miRNAs can either promote or impede oncogenesis by altering processes such as cell proliferation, invasion, angiogenesis, and apoptosis. Some overexpressed miRNAs manifest oncogenic properties, leading to ineffective tumour suppression mechanisms and uncontrolled cell division. Conversely, other miRNAs are downregulated, hindering their tumour suppressive properties [14].

Our study aimed to investigate the therapeutic potential of a synthetic chalcone compound, SHG-44, compared to standard first-line chemotherapy in pre-clinical U87MG and U251MG GB cell line models. We further sought to elucidate its cellular mechanisms of action by employing an array of functional assays alongside performing small RNA-sequencing (sRNAseq) to assess its action upon the miRNome of GB cells. We observed a substantial decrease in viability, migration and colony formation in U87MG and U251MG cells when exposed to SHG-44. The compound acted prominently upon the miRNA expression profiles within GB cell models.

## Methods

### Chalcone molecule (SHG-44) synthesis and characterisation

The reagents and solvents were purchased from Merck and Spectrochem and used without further purification unless otherwise mentioned. The 10% NaOH solution (2.0 ml) was added to a stirring mixture of 4-chloroacetophenone (**1**) (1 mol) and 4-methylbenzaldehyde (**2**) (1 mol) in 10 ml of ethanol at room temperature. The reaction mixture was stirred at room temperature for 15 min. After completion of the reaction as indicated by Thin Layer Chromatography (TLC), the reaction mixture was poured into crushed ice in mild acidic condition to neutralize sodium hydroxide in the reaction mixture. The chalcone (SHG-44) precipitated as a solid and then was filtered, washed with water, dried and recrystallized from ethanol to afford the pure compound SHG-44 as a light yellow crystalline solid with a yield of 85%. The progress of the reactions was monitored by using thin layer chromatography (TLC) on silica gel plates, and the spots were visualized under ultraviolet (UV) light (254nm). The infrared (IR) spectra’s of samples were recorded on a PerkinElmer FTIR, ATR mode spectrophotometer and data are reported in wave numbers (cm^−1^). The melting point for the synthesized compounds was determined by taking in the melting point capillary tube and by using the Stuart (SMP 11) melting point apparatus. The HRMS of the sample was performed on an Agilent 6540 HD Accurate Mass QTOF/LC/MS with electrospray ionization (ESI). ^1^H NMR and ^13^C NMR spectra were recorded using a Bruker Avance III HD 500 MHz NMR spectrometer. Proton chemical shifts were reported in ppm (δ) relative to internal tetramethylsilane (TMS, δ 0.00) or with the solvent reference relative to TMS as the internal standard (CDCl_3_, δ 7.26 ppm). The data obtained was reported as follows: chemical shift multiplicity [singlet (s) and doublet (d)], coupling constants [Hz], and integration. Carbon chemical shifts were reported in ppm (δ) relative to TMS with the respective solvent resonance as the internal standard (CDCl_3_, δ 77.0 ppm) and tetramethylsilane (TMS) as an internal standard.

### Cell culture and *in vitro* functional assays

Two commercially available human GB cell lines, U87MG and U251MG (ATCC, Manassas, VA, USA) were cultured in MEM (Minimum Essential Medium; GibcoTM, Bleiswijk, NL) supplemented with 10% foetal bovine serum (FBS; GibcoTM, Bleiswijk, NL) and 100 IU/mL penicillin-streptomycin (GibcoTM, Bleiswijk, NL) to promote growth. All GB cells were authenticated before the start of the experiments and experiments were performed with cells at parage numbers between 20–25. Cells were maintained at 37°C and 5% CO_2_. The 3-(4,5-dimethylthiazol-2-yl)-2,5diphenyltetrazolium bromide (MTT) protocol utilized was previously described by [15]. U87MG and U251MG cells were exposed to increasing concentrations of SHG-44 (20ΜM, 40ΜM, 60ΜM, 80ΜM, and 100ΜM), prepared as dilutions in Dimethylsulfoxide (DMSO, ThermofisherTM, CA, USA). Control wells were treated with 1% DMSO (negative control) or cis-platin as a positive control (200ΜM for U87MG and U251MG; Sigma-AldrichTM, Dorset, UK). The IC_50_ of cis-platin was previously determined in the same GB cell lines and was found to be 200ΜM [15]. HEK293 cells, a normal human embryonic kidney cell line, were used as a control to assess the effect of SHG-44 on non-cancerous cells by being exposed to increasing concentrations of 50ΜM, 75ΜM, 100ΜM, 150ΜM, and 200ΜM SHG-44. The cells were cultured in MEM supplemented with 2mM glutamine (GibcoTM, Bleiswijk, NL), 1% non-essential amino acids (GibcoTM, Bleiswijk, NL), and 10% fetal calf serum (FCS, GibcoTM, Bleiswijk, NL). Control wells were treated as described above. Colony formation, scratch, and ROS assays, alongside acridine orange/ethidium bromide staining were performed as previously described [15]. Throughout these assays, U87MG cells were exposed to SHG-44 at two concentrations (70.39ΜM and 100ΜM), 1% DMSO, and 200ΜM cis-platin. The concentrations of SHG-44 for U251MG cell treatment were 50ΜM and 100ΜM, 1% DMSO, and 200ΜM cis-platin. Furthermore, colony formation and wound healing assays were performed to compare the effects of SHG-44 and cis-platin on GB cells, using an equal concentration of 100ΜM for both compounds.

### 4′,6-diamidino-2-phenylindole, dihydrochloride staining

U87MG and U251MG cells were seeded at a density of 3 x 10^4^ cells/well. Following overnight incubation, cells were exposed to 1% DMSO (negative control), 200ΜM cis-platin, and SHG-44 (70.39ΜM and 100ΜM for U87MG; 50ΜM and 100ΜM for U251MG) for 24h. After treatment, the culture media was aspirated, and cells were washed twice with PBS. Cells were then fixed with 4% PFA for 15 min at room temperature, followed by two additional PBS washes. To visualize nuclei, cells were counterstained with DAPI (4’,6-diamidino-2-phenylindole, dihydrochloride; ThermofisherTM, CA, USA) mounting media and coverslips were mounted onto the slides. Immunofluorescence images were captured using a 40x objective on an EVOS fluorescent microscope (Life Technologies, CA, USA) with a pre-set exposure of 30 lx/s.

### RNA extraction and small RNA-sequencing

U87MG RNA samples were extracted as described by Braoudaki *et al*. (2016) [16]. In brief, total RNA of 1% DMSO and SHG-44 (100ΜM) 24h treated cells were extracted with Trizol reagent (Ambion Life Technology, Aukland, New Zealand). A nanodrop bioanalyzer was used to detect the concentration of each sample (Nanodrop ND1000 Spectrophotometer, Marshall Scientific, Hampton, USA). RNA integrity was assessed using the RNA Screen Tape Kit in the Agilent Bioanalyzer 2100 system (Agilent Technologies, CA, USA). Samples with RNA integrity number

(RIN) numbers greater than seven were sent off for external sRNA-seq to Biomarker Technologies (Biomarker Technologies, Inc., CA, USA). Small RNA library preparation utilized 1.5 Μg RNA per sample via using the NEBNext® Ultra™ Illumina kit (NEB, USA). This involved 3’ and 5’ adapter ligation and reverse transcription. Finally, PCR amplification and size selection enriched for small RNA libraries. Libraries were then clustered and sequenced on an Illumina HiSeq platform. Raw data was processed to remove low-quality reads and filter for lengths between 18–30 nucleotides. Clean reads were used for downstream analyses. Reads were aligned against databases to remove rRNA, tRNA, and other non-coding RNAs. Known and novel miRNAs were identified. Differential expression analysis was performed to compare miRNA levels between the DMSO and SHG-44 groups. Gene functions of miRNA targets were annotated using databases like Nr (NCBI non-redundant protein sequences: https://www.ncbi.nlm.nih.gov/refseq/); Nt (NCBI non-redundant nucleotide sequences: https://www.ncbi.nlm.nih.gov/refseq/) Pfam (Protein family: http://pfam.xfam.org); KOG/COG (ClustersofOrthologousGroupsof proteins: http://www.pdg.cnb.uam.es/cursos/Leon2002/pages/software/DatabasesListNAR2002/summary/7.html#:~:text=Database%20Description,orthologs%20(direct%20evolutionary%20counterparts); Swiss-Prot (A manually annotated and reviewed protein sequence database: https://www.uniprot.org); KO (KEGG Ortholog database: https://www.genome.jp/kegg/ko.html); GO (Gene Ontology: https://geneontology.org).

### RT-qPCR expression analysis

MiRNA RT-qPCR was performed as previously outlined by our team [15]. Briefly, total RNA and miRNAs were extracted using the Trizol reagent (Ambion Life Technology, Auckland, New Zealand) protocol and the mirVana isolation kit (ThermoFisher, Vilnius, Lithuania), respectively. The concentration and quality of the samples were evaluated using a Nanodrop spectrophotometer (Nanodrop ND1000, Marshall Scientific, Hampton, USA). Each RT-qPCR experiment for miRNA expression analysis was performed in triplicate with TaqMan Universal PCR Master Mix (no UNG) (Applied Biosystems ThermoFisher, Pleasanton, CA) using three independent biological samples per cell line on 96-well PCR plates. MiR-RNU-44 was used as an internal control. RT-qPCR gene expression analysis using SYBR Green was performed with the PowerUp SYBR Green Mastermix (Applied Biosystems, Massachusetts, USA) for SYBR green qPCR analyses. The primer sequences used for SYBR Green assays were as follows: *GAPDH* forward (GGAGCGAGATCCCTCCAAAAT) and reverse (GGCTGTTGTCATACTTCTCATGG), *Bcl2* forward (ATCGCCCTGTGGATGACTGAGT) and reverse (GCCAGGAGAAATCAAACAGAGGC), and *CAS6* forward (GCAGATCCCGGAATACAGTT) and reverse (GCTTGAGTTGGCAGAGGTTC).

### *In silico* analysis of miRNAs, SHG-44 and its metabolites

To identify the expression profiles of selected miRNAs, we utilized the dbDEMC database (https://www.biosino.org/dbDEMC/index), a comprehensive online resource that compiles differentially expressed miRNAs across various cancer types based on high-throughput sequencing and microarray data [17]. To explore the functional relevance of these miRNAs in GB, KEGG pathway and Gene Ontology (GO) analyses were conducted using the miRPath v4.0 database (http://62.217.122.229:3838/app/miRPathv4), which enables pathway enrichment analysis based on experimentally validated and predicted miRNA targets [18]. Additionally, Sfold binding site prediction analysis was performed to assess potential miRNA–mRNA interactions, specifically focusing on the identification of seed region binding sites and hybridization energies between selected miRNAs and their target genes (https://sfold.wadsworth.org/cgi-bin/index.pl) [13]. The metabolic properties of SHG-44 and its metabolites were predicted using Biotransformer 3.1.0 via employing strings to simulate metabolic transformations (canonical Simplified Molecular-Input Line-Entry System (SMILES) [19]. ADMET properties, such as absorption, distribution, metabolism, excretion and toxicity for SHG-44 and its metabolites, were then predicted with admetSAR 2.0, a machine learning tool that utilizes data from 96,000 compounds. The “ADMET properties for drug discovery” option was chosen within admetSAR to tailor the predictions towards a drug discovery context [20].

### Statistics

All statistical analyses were performed using GraphPad Prism version 9.4.1 software (GraphPad Software, San Diego, USA). This software was also used to generate all the figures presented in this study. Unpaired t-tests were employed to assess statistical differences between two groups, while one-way ANOVA/two-way ANOVA followed by Tukey’s multiple comparisons post-hoc test was used for comparisons among three or more groups. A p-value of less than 0.05 was considered statistically significant.

### Ethics statement

This study did not require ethical approval as there were no patients or animals involved in the research work outlined.

## Results

### Synthesis and characterisation of (*E*)-1-(4-chlorophenyl)-3-(ptolyl)prop-2-en-1-one (SHG-44)

**Fig 1:**
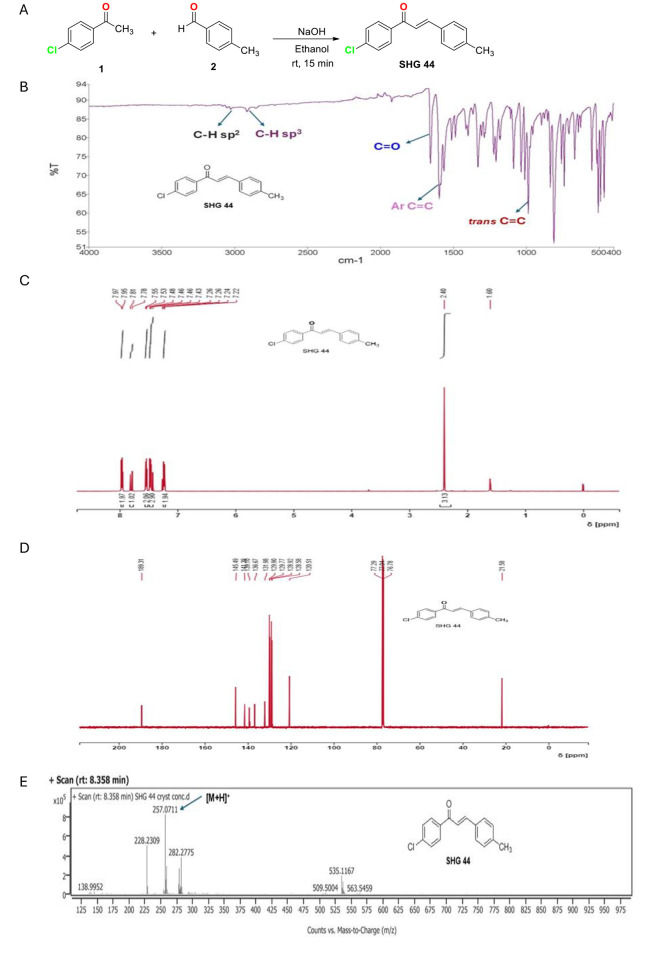
Synthesis and characterization of SHG-44. **(A)** Synthesis of **(*E*)**-1-(4-chlorophenyl)3-(p-tolyl)prop-2-en-1-one (SHG-44). **(B)** FTIR spectrum of SHG-44. **(C)** ^1^H NMR spectrum in CDCl_3_ and **(D)** ^13^C NMR spectrum CDCl_3_. **(E)** High-resolution mass spectrum (HRMS).

The (*E*)-1-(4-chlorophenyl)-3-(p-tolyl)prop-2-en-1-one (SHG-44) was synthesized by a conventional method using the Claisen-Schmidt condensation reaction [21]. In this process, the condensation of 4-chloroacetophenone (**1**) and 4-methylbenzaldehyde (**2**) was carried out in an ethanol solvent and addition of sodium hydroxide solution at room temperature and in ethanol solvent to yield a chalcone molecule, SHG-44 (Fig 1A). The synthesized molecule was characterized by melting point, FT-IR, HRMS, ^1^HNMR and ^13^C NMR spectroscopic techniques. The observed melting point of SHG-44 was 160−165°C. In the FT-IR spectrum of SHG-44, a sharp peak at 1657 cm^-1^ confirms the presence of the C = O group (Fig 1B). The peaks in the IR region at 3026 and 2918 cm^-1^ (very weak) corresponded to C–H sp^2^ and C–H sp^3^, respectively. The observed peaks at 1599 (medium), and 988 (strong) cm^-1^ were assigned to aromatic C = C, and trans C = C vibrations, respectively. From the ^1^H- and ^13^C-NMR spectra analysis of SHG-44, the following chemical shifts were detected: ^1^H-NMR (500 MHz, CDCl_3_) δ/ppm: 2.40 (s, 3H, -CH_3_), 7.23 (d, 2H, J = 10 Hz, Ar–H), 7.44 (d, 1H, J = 15 Hz, C = C trans), 7.47 (d, 2H, J = 10 Hz, Ar–H), 7.54 (d, 2H, J = 10 Hz, Ar–H), 7.80 (d, 1H, J = 15 Hz, C = C trans), 7.94 (d, 2H, J = 10 Hz, Ar–H). ^13^C-NMR (CDCl_3_) δ/ppm: 21.58 (-CH_3_), 120.5 & 145.4 (C = C trans), 189.3 (C = O), 128.5, 128.9, 129.7, 129.9, 131.9, 136.6, 139.1, 141.3 (C aromatics) (Figs 1C–1D). In the HRMS (ESI) spectra, the m/z peak is found at 257.0711, matching with the calculated m/z for C_16_H_14_ClO [M + H]^+^, which was 257.0733, showing a delta value of 0.0022 (Fig 1E).

### SHG-44 treatment suppressed the proliferation of U251MG and U87MG cells

**Fig 2:**
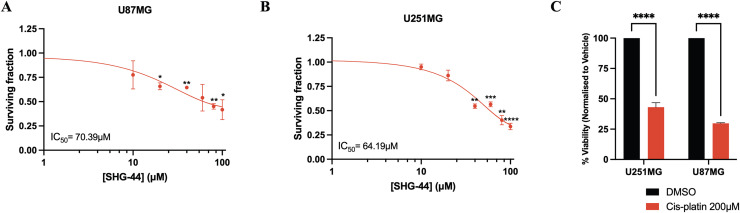
Reduction in cell viability of U87MG and U251MG glioma cells post exposure to SHG-44. (A) A significant reduction in U87MG cell viability compared to the DMSO control. (0ΜM SHG-44) with a half-maximal inhibitory concentration (IC_50_) of 70.39ΜM. **(B)** A significant reduction in U251MG cell viability compared to the DMSO control with a half-maximal inhibitory concentration (IC_50_) of 64.19ΜM. **(C)** Treatment with 200ΜM cis-platin (positive control) resulted in significantly reduced cell viability within both cell lines. The data represents mean values and standard deviation, n = 3. **Legends**: ns: non-significant, *p < 0.05, **p < 0.01, ***p < 0.001, ****p < 0.0001.

The cytotoxic potential of SHG-44 upon U87MG and U251MG GB cells demonstrated a significant reduction in cell viability when compared to the DMSO control (Figs 2A–2B). The tested concentrations of SHG-44 upon both cell lines were 20ΜΜ, 40ΜΜ, 60ΜM, 80ΜΜ and 100ΜM. The half-maximal inhibitory concentration (IC_50_) in U87MG cells was determined as 70.39ΜM (*p* < 0.05), whilst the compound exhibited greater cytotoxic capacity in U251MG cells with an IC_50_ of 64.19ΜM (*p* < 0.05). Additionally, both cell lines received treatment with 200ΜΜ cis-platin, which resulted in significantly reduced cell viability, comparable with what was seen in cells treated with half the dose of SHG-44 (*p* < 0.001) (Fig 2C). MTT assays performed on HEK293 cells demonstrated that the IC₅₀ of SHG-44 was considerably higher in these normal cells compared to GB cells. Specifically, the IC₅₀ values for HEK293 cells were 179.27ΜM at 24h (*p* < 0.0001) and 189.04ΜM at 48h (*p* < 0.0001), indicating a significantly lower sensitivity to SHG-44 (S1 Fig). In contrast, GB cells exhibited much lower IC₅₀ values, highlighting their increased susceptibility to the compound.

### SHG-44 impaired the colony-forming potential of U251MG and U87MG cells

**Fig 3:**
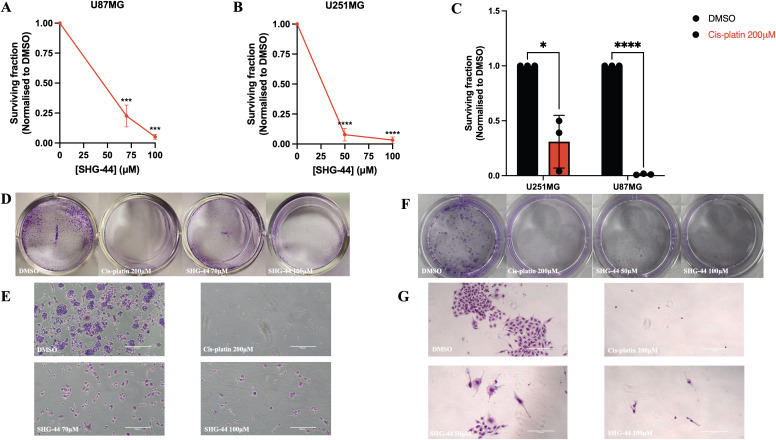
Decreased clonogenic capacity of U87MG and U251MG glioma cells dependent on SHG-44 concentration. **(A)** A significant decrease in the proliferative clonal capacity of U87MG colonies was observed with 70ΜM and 100ΜM SHG-44 treatment. **(B)** A significant decrease in the proliferative clonal capacity of U251MG colonies was observed with 50ΜM and 100ΜM SHG-44 treatment. **(C)** Cis-platin 200ΜM (positive control) also significantly inhibited the formation of colonies within both cell line models. **(D-E)** This effect was also evident microscopically, where inhibition of colony formation was observed in both U87MG and **(F-G)** U251MG cells. Microscopic images of colonies were taken at ×40 magnification. The data represents mean values and standard deviation, n = 3. **Legends:** ns: non-significant, *p < 0.05, **p < 0.01, ***p < 0.001, ****p < 0.0001.

The impact of SHG-44 on the U87MG and U251MG cells revealed a significant decrease in clonogenic potential in both GB cell lines (Figs 3A–3B). Cells were treated with 1% DMSO, cis-platin 200ΜM, 70ΜM and 100ΜM SHG-44 (U87MG), and 50ΜM and 100ΜM SHG-44 (U251MG). The lower test concentration for the U251MG cells was employed with the consideration that these cells were more susceptible to the compound, as demonstrated via the cell viability assay. Cells treated with cis-platin (200ΜM) also revealed significantly lower survival rates when compared to the control (Fig 3C). Compared to the DMSO control, treatment with 70ΜM and 100ΜM SHG-44 significantly reduced (*p* < 0.004, *p* < 0.0001, respectively) U87MG colony formation, with only 22% and 5% cell survival observed, respectively (Figs 3D–3E). The survival fraction of U251MG cells treated with 50ΜM and 100ΜM SHG-44 was also significantly reduced in comparison to DMSO (9% (*p* < 0.0001) and 3% (*p* < 0.0001), respectively) (Figs 3F–3G). Clonogenic assays were also performed using equal concentrations (100ΜM) of SHG-44 and cis-platin to directly compare their effects at same concentrations. SHG-44 treatment resulted in a significantly greater reduction in colony-forming ability in both U87MG and U251MG cells compared to cis-platin (S2 Fig). Microscopic imaging confirmed a near-complete absence of colonies following SHG-44 treatment, indicating a stronger inhibition of long-term proliferative potential by the SHG compound (S2 Fig).

### SHG-44 disrupted the migratory abilities of U251MG and U87MG cells

**Fig 4:**
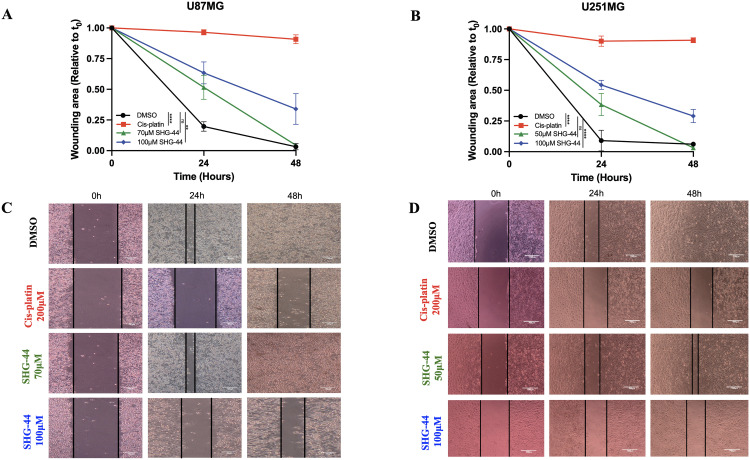
Inhibited cell migration of U87MG and U251MG glioma dependent on SHG-44 concentration. **(A)** Quantification of the wound-healing assay for U87MG cells following treatment with DMSO, cis-platin, and SHG-44 (70ΜM and 100ΜM) at various time points. **(B)** Wound-healing area quantification for U251MG cells following treatment with DMSO, cis-platin, and SHG-44 (50ΜM and 100ΜM) at various time points. **(C)** Scratch images were acquired at 0h, 24h, and 48h from U87MG cells treated as described above. **(D)** Scratch images were acquired at 0h, 24h, and 48h from U251MG cells treated as described above. Microscopic images of scratch areas were taken at ×40 magnification. The data represents mean values and standard deviation, n = 3. **Legends:** ns: non-significant, *p < 0.05, **p < 0.01, ***p < 0.001, ****p < 0.0001.

To assess the potential anti-migratory effects of SHG-44, wound healing assay was performed upon U87MG and U251MG GB cells treated with 1% DMSO, cis-platin (200ΜM), and increasing concentrations of SHG-44 (70ΜM and 100ΜM in U87MG, and 75ΜM and 100ΜM in U251MG). The wound area was quantified at 0h, 24h, and 48h post-treatment (Figs 4A–4B). Cis-platin treatment significantly inhibited wound closure compared to the DMSO control in both cell lines (*p* < 0.0001). Treatment with 70ΜM SHG-44 exhibited wound closure rates similar to the DMSO control at 24h and 48h, respectively (*p* < 0.7577) (Fig 4C). However, 100ΜM SHG-44 significantly impaired wound closure at 24h and 48h, compared to the control (*p* < 0.01) (Fig 4C). Similarly, treatment with 50ΜM SHG-44 did not show an impairment of cell migration when compared to DMSO (24h and 48h), whilst 100ΜM SHG-44 seized the migratory abilities of U251MG cells significantly at these time points (*p* < 0.9981, *p* < 0.001, respectively, Fig 4D). Wound healing assays were also performed using SHG-44 and cis-platin at equal concentrations (100ΜM) to assess their effects on cell migration at same concentrations. Both treatments significantly reduced migration in U87MG and U251MG cells compared to the DMSO control (S3 Fig). No significant difference in wound closure was observed between SHG-44 and cis-platin, suggesting that both agents exert comparable anti-migratory effects under these conditions (S3 Fig).

### SHG-44 exhibited no ROS-inducing effects

**Fig 5:**
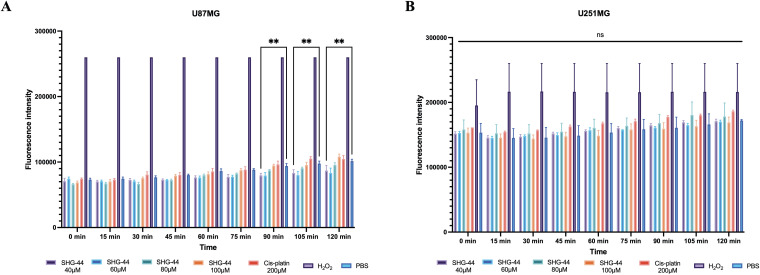
SHG-44 did not lead to a significant production of ROS release. **(A)** A nonconcertation dependent release of ROS was observed only at 40ΜM SHG-44 concentration within U87MG cells after 90 min incubation. **(B)** There was no significant release of ROS at any time point and any SHG-44 concentration within U251MG cells. The data represents mean values and standard deviation, n = 3. **Legends:** ns: non-significant, *p < 0.05, **p < 0.01, ***p < 0.001, ****p < 0.0001.

To evaluate the potential effects of SHG-44 on cellular ROS levels in U87MG and U251MG cells, ROS was quantified after treatment with PBS (negative control), cis-platin (200ΜM), and increasing concentrations of SHG-44 (40ΜM, 60ΜM, 80ΜM, and 100ΜM). Interestingly, treatment with high SHG-44 concentrations (60ΜM, 80ΜM and 100ΜM) did not affect ROS levels compared to the control within U87MG cells. However, U87MG cells treated with 40ΜM SHG-44 had significantly increased ROS levels (*p* < 0.0001) (Fig 5A). Meanwhile, the ROS analysis of U251MG cells did not show significant elevation of ROS levels at any SHG-44 concentration (Fig 5B).

### SHG-44 mediated apoptosis in U87MG and U251MG glioma cells

**Fig 6:**
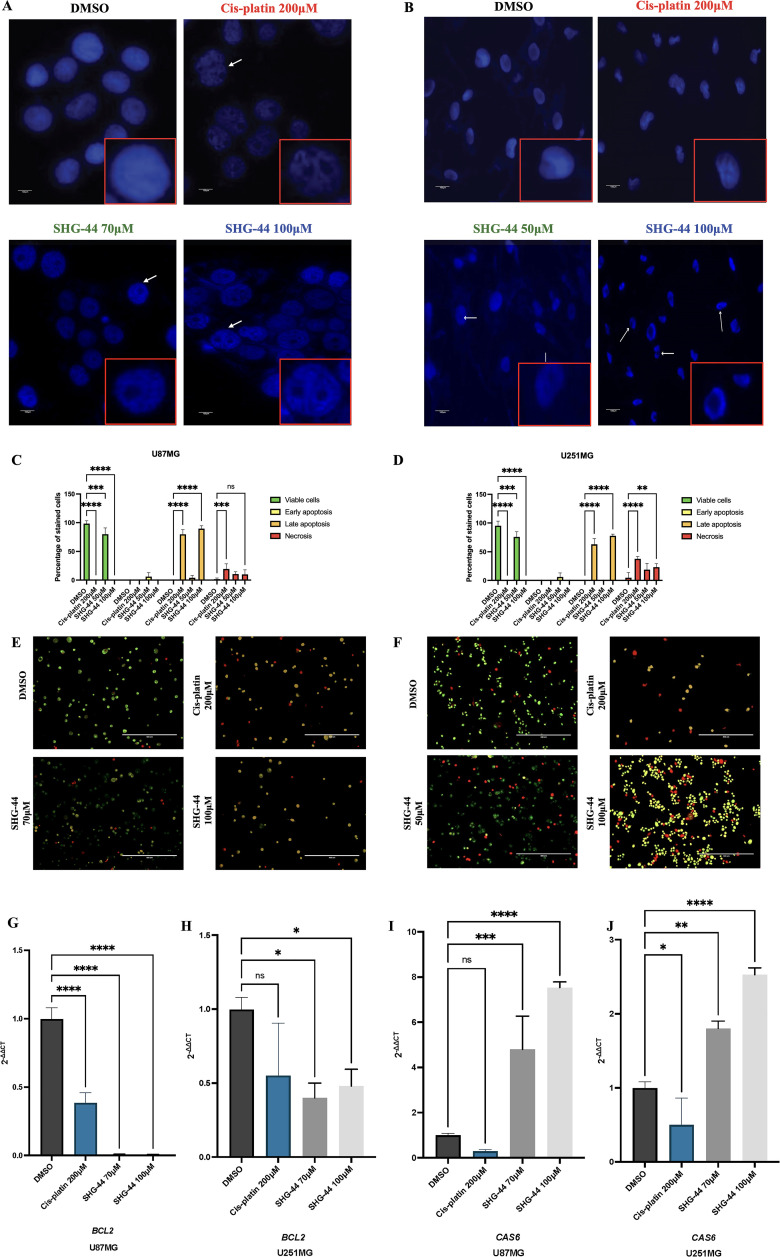
SHG-44 leads to disruptions in the cell nucleus morphology and apoptosis. **(A)** DAPI staining images demonstrated nuclear fragmentation following treatments with SHG-44 (70ΜM and 100ΜM) within U87MG cells**. (B)** DAPI staining images demonstrated nuclear fragmentation following treatments with SHG-44 (50ΜM and 100ΜM) within U251MG cells (white arrows indicate apoptotic cell morphology characterized by nuclear fragmentation in U87MG and U251MG cells). **(C)** Quantification of apoptosis within U87MG cells post exposure to DMSO, 200ΜM cis-platin, and 70ΜM and 100ΜM SHG-44. **(D)** Quantification of apoptosis within U251MG cells post exposure to DMSO, 200ΜM cis-platin, and 50ΜM and 100ΜM SHG-44. **(E)** Fluorescent microscopic images of U87MG GB cells treated with DMSO, 200ΜM cis-platin, and 70ΜM and 100ΜM SHG-44. **(F)** Fluorescent microscopic images of U251MG GB cells treated with DMSO, 200ΜM cis-platin, and 50ΜM and 100ΜM SHG-44. Green cells indicate viability, yellow cells indicate early apoptosis, orange cells indicate late apoptosis, and red cells indicate necrosis. **(G-H)** *Bcl2*, an anti-apoptotic gene, showed significant downregulation in both U87MG and U251MG cells, with a more pronounced effect at the 100ΜM concentration. **(I-J)** *CAS6*, a pro-apoptotic effector gene, was upregulated in both cell lines following SHG-44 treatment, indicating an activation of apoptotic pathways. The data represents mean values and standard deviation, n = 3. **Legends:** ns: nonsignificant, *p < 0.05, **p < 0.01, ***p < 0.001, ****p < 0.0001.

To investigate the potential involvement of apoptosis induction in the cytotoxic action of SHG-44, U87MG and U251MG cells were treated 1% DMSO, cis-platin (200ΜM), and increasing concentrations of SHG-44 (70ΜM and 100ΜM within U87MG, and 50ΜM and 100ΜM within U251MG). Subsequently, cells were stained with DAPI for visualization of nuclear morphology and assessment of treatment-induced changes (Figs 6A–6B). DMSO-treated U87MG and U251MG cells displayed typical intact nuclei. In contrast, treatment with cis-platin or either concentration of SHG-44 resulted in prominent nuclear fragmentation within both cell lines, characterized by multiple nuclear ruptures. To further determine the effects of SHG-44 on apoptosis and Acridine Orange/Ethidium Bromide (AO/EtBr) staining was utilized to assess cell death U87MG and U251MG cells (Figs 6C–6F). Treatments with 100ΜM SHG-44 resulted in high ethidium bromide uptake, indicating a loss of membrane integrity and cell death. At 50ΜM SHG-44 appeared to induce similar levels of early apoptosis in both, U87MG and U251MG cells. Similar apoptotic and necrotic cell death was observed with 200ΜM cis-platin treatment. The negative control (DMSO) showed no signs of drug induced cell death, with cells maintaining an intact and regular shape. To investigate the apoptotic effects of SHG-44 on GB cell lines, U87MG and U251MG cells were treated and subsequently analyzed for the expression of key apoptosis-related genes, specifically *Bcl2* and *CAS6*. Quantitative RT-qPCR analysis revealed significant modulation of these genes in response to SHG-44 treatment at 70ΜM and 100ΜM concentrations, with the higher concertation being more effective in the dysregulation of gene expression. *Bcl2*, an anti-apoptotic gene, was downregulated in both U87MG and U251MG cells post-treatment (Figs 6G–6H), whilst *CAS6* (caspase-6), a pro-apoptotic effector gene involved in the execution phase of apoptosis, was upregulated in both cell lines following treatment with SHG-44 (Figs 6I–6J).

### SHG-44 influenced cellular functions through its action upon the U87MG’s miRNome

**Fig 7:**
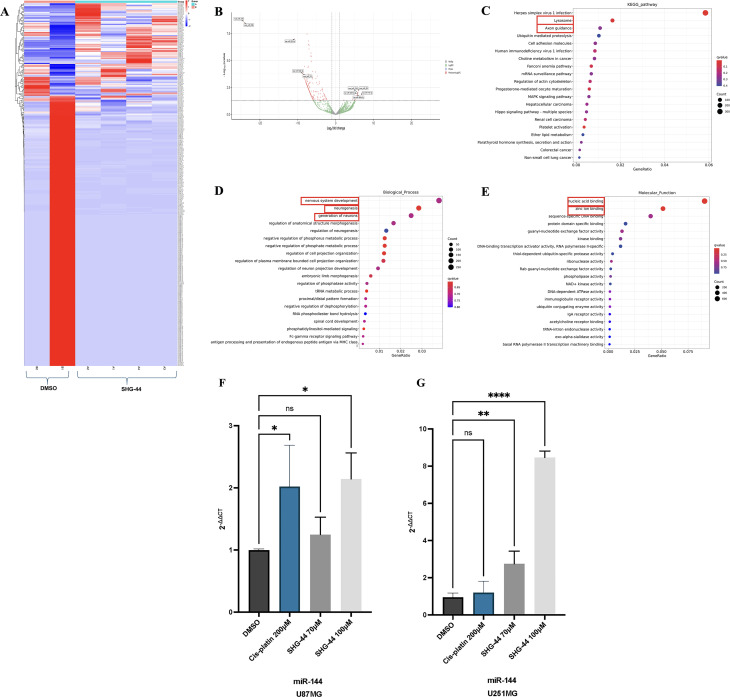
Dysregulation of miRNAs following treatment with SHG-44. **(A)** Heatmap displaying 254 dysregulated miRNAs (231 downregulated and 23 upregulated). **(B)** Volcano plot highlighting the top five most upregulated and downregulated miRNAs. The most downregulated miRNAs were miR-122b-5p and miR-223-3p, while the most upregulated were miR-144-3p, miR-5009-5p, and miR-664b-3p, excluding novel miRNAs. **(C)** KEGG Pathway analysis revealed that the dysregulated miRNAs in the presence of SHG-44 play significant roles in pathways such as axon guidance, cell adhesion, and ubiquitin-mediated proteolysis. **(D)** Biological process analysis indicated that most dysregulated miRNAs were involved in nervous system development, neurogenesis, and the generation of neurones. **(E)** Molecular function analysis revealed that the dysregulated miRNAs were involved mainly in nucleic acid binding and zinc ion binding. **(F)** In U87MG cells, miR-144-3p expression was significantly upregulated at both concentrations, with a more pronounced increase observed at 100ΜM. **(G)** Similarly, U251MG cells showed significant upregulation of miR-144-3p following treatment at both 70ΜM and 100ΜM, paralleling the response seen in U87MG cells. The data represents mean values and standard deviation, n = 3. **Legends:** ns: nonsignificant, *p < 0.05, **p < 0.01, ***p < 0.001, ****p < 0.0001.

The sRNA-seq data revealed that treatment with 100ΜM SHG-44 significantly altered the miRNA expression profiles in U87MG cells. The heatmap in Fig 7A illustrated that a total of 254 miRNAs were deregulated in the presence of SHG-44, with prevenance towards downregulation (231) compared to upregulation (23). The most dramatic changes involved the downregulation of miR122b-5p and miR-223-3p and the upregulation of miR-144-3p, miR-5009-5p, and miR-664b-3p (Fig 7B). Further analyses (Figs 7C–7E) suggested that the deregulated miRNAs played a role in specific signallingpathways, such as axon guidance, protein degradation, and cell adhesion. Interestingly, the deregulated miRNAs also appeared to influence various biological processes, such as nervous system development, neurogenesis, generation of neurones and regulation of neurogenesis. At the molecular level, they were seen to interact with targets involving nucleic acids, zinc ions, sequence-specific DNA binding, and protein domain-specific binding. These findings suggested that SHG-44 has the potential to broadly influence cellular functions through its effects on miRNA expression. To validate our sRNA-seq results, RT-qPCR was performed to assess the expression levels of miR-144-3p in U87MG and U251MG GB cells following treatment with SHG-44 at concentrations of 70ΜM and 100ΜM. In U87MG cells, RT-qPCR analysis confirmed that both concentrations of SHG-44 upregulated miR-144-3p expression, with the 100ΜM treatment showing a more pronounced increase in miR-144 levels compared to the 70ΜM treatment (Fig 7F). Similarly, in U251MG cells, treatment with both 70ΜM and 100ΜM SHG-44 led to significant upregulation of miR-144, mirroring the trend observed in U87MG cells (Fig 7G).

### SHG-44 possessed similar pharmacokinetic and pharmacodynamic characteristics to aspirin and ibuprofen

**Fig 8:**
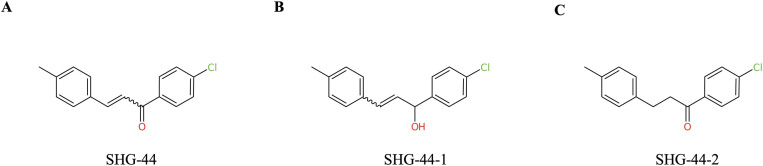
Metabolites of the parent compound SHG-44. **(A)** Skeletal formula of parental SHG-44 compound. **(B)** Metabolite SHG-44-1 is produced by carbonyl reductase. **(C)** Metabolite SHG-442 is produced by the reduction of alpha,beta-unsaturated carbon-carbon double bond adjacent to electron withdrawing group.

The two predicted metabolites (SHG-44–1 and SHG-44–2) were obtained based on the reduction of the parent compound SHG-44 (Fig 8A) by carbonyl reductase (Fig 8B), as well as the reduction of α,β-unsaturated carbon-carbon double bond adjacent to electron withdrawing group (Fig 8C). Selected absorption, distribution, and toxicity properties of SHG-44 and its metabolites, predicted using admetSAR 2.0, are shown in Table 1. For comparison, the over-the-counter painkillers aspirin and ibuprofen were included in the prediction. As seen in Table 1, SHG-44 had absorption, oral bioavailability, and distribution characteristics comparable to aspirin and ibuprofen, indicating that SHG-44 is likely to be well absorbed and distributed in the body. Notably, SHG-44 and its metabolites were predicted to pass the blood-brain barrier. SHG-44 and all metabolites fell into acute oral toxicity class III (medium), with class IV being the lowest toxicity class. In terms of various toxicity predictions, SHG-44 had a nephrotoxicity profile similar to aspirin, a mitochondrial toxicity profile similar to both aspirin and ibuprofen and reproductive and respiratory toxicity profiles differing from both aspirin and ibuprofen. SHG-44 was not associated with carcinogenicity and was negative for the micronucleus test. However, several metabolites were predicted to be positive in the hERG inhibition, a gene encoding the alpha-subunit of a cardiac potassium channel, which could potentially lead to heart problems. None of the SHG-44 metabolites were predicted to be carcinogenic in the Ames test.

**Table 1:** Prediction of absorption, distribution, and toxicity properties of SHG-44 and its metabolites, alongside predictions for aspirin and ibuprofen.

	Compound				
Feature^1^	SHG- 44	SHG- 44−1	SHG- 44−2	Aspirin	Ibuprofen
Caco-2 permeability	+	+	+	–	+
HumanIntestinal Absorption	+	+	+	+	+
Humanoral bioavailability	+	+	+	+	+
Blood Brain Barrier	+	+	+	–	+
P-glycoprotein inhibitior	+	+	–	–	–
P-glycoprotein substrate	–	–	–	–	–
Plasmaprotein binding^2^	100%	100%	92%	58%	75%
Ames mutagenesis	+	+	–	–	–
Acute Oral Toxicity^3^	III	III	III	II	III
Carcinogenicity (binary)	–	–	–	–	–
Micronucleus test	–	–	–	+	–
hERG inhibition	+	–	+	–	–
Mitochondrial toxicity	–	+	+	+	+
Nephrotoxicity	+	+	+	+	–
Reproductive toxicity	–	–	+	+	+
Respiratory toxicity	–	–	+	+	+

A ‘+’ symbol indicates a positive prediction or presence of a feature, while a ‘-’ symbol indicates a negative prediction or absence of a feature. The numbers are represented as percentages. The toxicity classes range from I to IV, with class IV representing the lowest possible toxicity.

## Discussion

Clinically actionable combination therapies for GB often exhibit promising efficacy in reducing resistant tumour clones [4]. However, their clinical application is hampered by the frequent occurrence of severe side effects and significant financial burden. Consequently, there is growing interest in natural and synthetic chalcones due to their reported low toxicity and multifaceted pharmaceutical properties, although their mechanisms of action remain largely unexplored [22]. This study aimed to address this gap in knowledge by investigating the therapeutic potential of the synthetic chalcone, SHG-44, in U87MG and U251MG GB cell line models. Furthermore, we sought to elucidate the multifaceted mechanisms of action of SHG-44 at the cellular and molecular levels.

Chalcone compounds, characterized by a 1,3-diaryl-2-propen-1-one skeleton, have emerged as potential candidates for cancer therapy due to their diverse biological activities [23]. These properties include induction of apoptosis, modulation of cell cycle progression, and inhibition of angiogenesis. *In vitro* studies have demonstrated promising cytotoxic effects of chalcones against various cancer cell lines, including those derived from breast, colon, and lung cancers [24,25]. Despite these encouraging findings, further in vivo studies and clinical trials are necessary to fully assess the therapeutic efficacy and safety profile of chalcones for cancer treatment.

The majority of reported chalcone molecules for GB targeting are characterized with polar substituents such as hydroxyl (OH), amine (NH₂) or methoxy (OCH₃) groups on the aromatic or heterocyclic ring of chalcone framework [26,27]. These polar groups are known to interact favorably with biological targets, potentially contributing to the activity of these molecules. In contrast, SHG-44 is a chalcone derivative with hydrophobic methyl (CH₃) and weakly polar chlorine (Cl) group on chalcone aromatic rings introducing new chemical diversity compared to the more commonly used polar groups. Therefore, SHG-44 could possess the ability to target hydrophobic pockets in GB-related proteins, potentially enhancing drug permeability and selectivity. This structural change is novel, as it has not been widely explored in chalcone compounds used against GB cells. The implication of these differences between existing chalcones and SHG-44 opens possibilities for optimizing the drug-like properties of chalcone derivatives for GB targeting, highlighting the potential of the newly synthesized SHG-44 to enhance current therapeutic strategies in GB treatment.

Our research demonstrated that SHG-44 treatment significantly reduces cell viability, colony formation, and migration, suggesting its potential as an anti-tumour agent against GB. The chalcone molecule, SHG-44 exhibited dose-dependent cytotoxicity against both U87MG and U251MG cells, with lower IC_50_ within the later cells, suggesting a better efficacy against U251MG cells. The observed reduction in cell viability aligns with previous studies on chalcone derivatives, which have been shown to induce cell death in various cancers [27–29]. Furthermore, the significantly higher IC₅₀ values observed in HEK293 cells suggested that SHG-44 potentially exhibits a greater cytotoxic effect upon GB cells when compared to normal cells. This differential sensitivity indicates a possible therapeutic window where SHG-44 could effectively target GB while minimising toxicity to non-cancerous cells. In addition, SHG-44 treatment significantly impaired the colony-forming potential of both cell lines. This indicated that SHG-44 not only inhibited existing cell proliferation but also disrupted the ability of GB cells to form new colonies, potentially hindering tumour growth. SHG-44 exposure impeded cell migration in U251MG and U87MG cells, thus suggesting that the compound may target cellular processes crucial for tumour metastasis. When tested at an equivalent concentration, SHG-44 demonstrated significantly stronger anti-tumorigenic activity than cis-platin in colony formation assays (S2 Fig), while both compounds showed similar efficacy in inhibiting cell migration (S3 Fig). These findings suggest that SHG-44 may offer better or similar therapeutic benefits compared to cis-platin, particularly in suppressing tumour growth.

While the exact mechanisms of how SHG-44 induces its inhibitory properties require further investigation, some studies have suggested that chalcones can trigger ROS production in cancer cells [30,31]. SHG-44 treatment elicited contrasting effects on ROS levels in U87MG and U251MG cells, displaying a cell line-specific response. In U87MG cells, high concentrations of the compound did not induce ROS production. However, lower concentrations of SHG-44 led to a significant release of ROS. This unexpected decrease in ROS levels at high concentrations suggested a potential activation of antioxidant pathways by SHG-44 at these concentrations within U87MG cells. Conversely, U251MG cells did not exhibit a significant ROS elevation upon SHG44 treatment, suggesting a potentially ROS-independent cytotoxic mechanism in this cell line. These observations deviate from the common description of chalcones as ROS-inducing agents [31]. It is important to note that ROS production might not be a universal effect of chalcones on GB cells. Potential exploration of specific antioxidant enzymes or signalling pathways activated by SHG-44 in each cell line could provide extra details of the specific mechanisms underlying the action of SHG-44 and the differential ROS responses within GB cell line models.

Current research has demonstrated the pro-apoptotic potential of chalcones in various cancer types, including ovarian, oesophageal, and breast cancers [32–34]. There is exciting evidence that synthetic chalcones have the potential to lead to cell death via apoptosis within GB cells [10,35]. Mechanistically, chalcones may induce apoptosis through diverse pathways, including modulation of Bcl-2 protein family members, activation of caspases, and disruption of mitochondrial function [36]. Our DAPI staining revealed a clear signature of apoptosis in both cell lines treated with SHG44 compared to the control. This finding, characterized by prominent nuclear fragmentation, suggested that SHG-44 triggers programmed cell death in glioma cells.

Further evidence supporting apoptosis induction was demonstrated by the AO/EtBr staining. Treatment with a higher concentration of SHG-44 resulted in increased ethidium bromide uptake by both cell lines, indicating a loss of membrane integrity and late-stage apoptosis or necrosis. Interestingly, 50ΜM SHG-44 appeared to induce early apoptosis in more U251MG cells compared to U87MG cells, suggesting potential differences in apoptotic susceptibility between the two lines. This observation aligns with previous studies demonstrating cell line-specific differences between the U87MG and U251MG cells [37]. Therefore, exploring the potential variations between different glioma cell lines and understanding the precise mechanisms of action could facilitate the individual tailoring of SHG-44 for targeted glioma therapy. The reciprocal expression pattern that we observed, with *Bcl2* downregulated and *CAS6* upregulated, indicates that SHG-44 treatment could promote an apoptotic response in GB cells, reducing anti-apoptotic defenses while activating genes involved in apoptotic execution. These findings support a potential apoptotic mechanism of action for SHG-44 in GB therapy.

We further examined the effects of SHG-44 on the miRNA regulatory network within GB cells, as previous research demonstrated that chalcone compounds could modulate miRNA activity in cancer. TChal, a small molecule trans-chalcone, has presented anti-tumour effects in breast cancer [38]. Treatment with TChal altered the expression of smiR-27a-3p, miR-449a, miR-25-5p, miR-4485, and miR-891a-5p. Specifically, miR-27a-3p and miR-449a were found to inhibit genes related to metastasis and proliferation, while miR-4485 targeted a gene linked to mitochondrial function and apoptosis [38].

Our sRNA-seq analysis revealed that the two most downregulated miRNAs in the presence of SHG-44 were miR-122b-5p and miR-223-3p, whilst the three most upregulated were miR-144-3p, miR-5009-5p and miR-664b-3p.

To the best of our knowledge, miR-122b-5p has not been previously linked to GB or other brain tumours. Our *in silico* expression analysis revealed that miR-122b-5p is highly overexpressed in GB patient samples when compared to controls, suggesting its potential role as an oncomiR in GB (S1 Table). Consistent with this, our sRNA-seq data showed that SHG-44 treatment led to a significant downregulation of miR-122b-5p, which may represent a key therapeutic mechanism of the drug. KEGG pathway analysis further identified miR-122b-5p as the only differentially expressed miRNA involved in apoptosis-related pathways, such as the p53, PI3K-Akt, and autophagy pathways (S2 Table). MiR-122b-5p was found to interplay key apoptosis-related genes including *BCL2*, *CASP3*, and *FAS* (S2 Table). To further support this, we performed a detailed analysis of miR-122b-5p target genes across these KEGG pathways and identified a total of 24 apoptosis-related targets, of which 20 were classified as pro-apoptotic and only 4 as anti-apoptotic (S4 Table). These genes were distributed across the apoptosis, PI3K-Akt, p53, and autophagy pathways (S2 File) and included central regulators such as *CASP3, TNFRSF1A, BID, APAF1, PTEN, FOXO3,* and *BNIP3*, indicating that miR-122b-5p may act to broadly suppress apoptotic signalling in GB states. Thus, based on these findings we could suggest that miR-122b-5p is frequently upregulated in GB, where it may contribute to tumorigenesis by suppressing pro-apoptotic pathways and enabling cancer cell survival. Our RT-qPCR data confirmed the upregulation of such pro-apoptotic target genes upon SHG-44 treatment, supporting a potential mechanism where SHG-44 promotes apoptosis by repressing miR-122b-5p. Furthermore, GO analysis of miR-122b-5p target genes revealed significant enrichment in terms related to protein binding, cytoplasmic localization, and cytosolic processes, suggesting a broad regulatory role in intracellular signalling and protein interaction networks (S3 Table). Taken together, our data suggested that SHG-44-induced downregulation of miR-122b-5p may relieve repression on key apoptotic genes, thus restoring programmed cell death in GB cells and contributing to the anti-tumour efficacy of the compound.

The second most downregulated miRNA following SHG-44 treatment was miR-223-3p. Our *in silico* analysis revealed that miR-223-3p is significantly upregulated in GB patient samples (S1 Table), suggesting a potential oncogenic role. Therefore, its downregulation by SHG-44 may represent a favourable therapeutic effect, contributing to the suppression of tumour-promoting pathways. MiR-223-3p has been previously associated with GB. A recent study has identified miR-223-3p as one of the most upregulated miRNAs in GB stem-like cells, further reinforcing its potential role as an oncomiR [39]. In addition, our KEGG pathway analysis revealed that miR-223-3p is involved in multiple cancer-related pathways, including the FoxO signalling pathway, signalling pathways regulating pluripotency of stem cells, and longevity regulating pathways (S2 Table). Our GO analysis showed enrichment in terms related to protein binding, nucleus, and cytoplasm, indicating potential involvement in key intracellular regulatory functions (S3 Table). The precise mechanisms by which miR-223-3p contributes to GB pathogenesis remain unclear. However, our data provide new insight into its oncogenic potential and indicate that its downregulation by SHG-44 may contribute to the anti-tumour properties of the compound in GB.

One of the three most upregulated miRNAs in response to SHG-44 treatment was miR-144-3p. Interestingly, our *in silico* expression analysis revealed that miR-144-3p is also overexpressed in GB patient samples when compared to controls, suggesting an oncogenic role (S1 Table). Thus, its further upregulation by SHG-44 may appear to be an unfavourable effect. However, previous studies have shown that downregulation of miR-144-3p in GB correlates with increased expression of *c-Met*, a receptor tyrosine kinase known to promote tumour proliferation, invasiveness, and poor prognosis [40]. This suggests that miR-144-3p may actually function as a tumour suppressor in this context. To explore this interaction, we performed Sfold binding site prediction analysis, which identified a single binding site for miR-144-3p within the 3′ UTR of *c-Met*, characterized as a 7mer-A1 site with a hybridization energy of –15.200 kcal/mol (S4 Fig), supporting the hypothesis of direct regulation. In addition, miR-144-3p has been shown to suppress cell proliferation and invasion in HCMV-positive GB by targeting *TOP2A*, a well-known biomarker in cancer progression and therapy [41]. Our Sfold analysis confirmed the presence of a binding site for miR-144-3p within the 3′ UTR of *TOP2A*, also a 7mer-A1 site, with a hybridization energy of –14.500 kcal/mol (S4 Fig). Furthermore, our *in silico* KEGG pathway analysis revealed that miR-144-3p is involved in several key regulatory pathways, including axon guidance, TGF-β signalling, and focal adhesion (S2 Table). Gene ontology analysis further indicated that miR-144-3p is primarily associated with positive regulation of transcription by RNA polymerase II, nucleoplasm, and nucleus, suggesting a role in transcriptional control and nuclear regulatory processes relevant to cancer biology (S3 Table). Together, these findings suggest that miR-144-3p may exert tumour-suppressive effects in GB, and its upregulation by SHG-44, while initially counterintuitive, may contribute positively to the anti-cancer activity of the compound.

MiR-5009-5p was among the most upregulated miRNAs following SHG-44 treatment. To the best of our knowledge, this miRNA has not previously been associated with brain tumours, and there is currently no evidence of its expression in GB according to data from the dbDEMC database. However, KEGG pathway analysis revealed that miR-5009-5p may participate in several cancer-related processes, such as chronic myeloid leukaemia, pathways in cancer, and axon guidance (S2 Table). Additionally, GO analysis suggested that miR-5009-5p is primarily associated with protein binding, nucleus, and cytoplasm, indicating potential involvement in intracellular signalling and regulatory functions (S3 Table). While its function in cancer remains unclear, miR-5009-5p downregulation has been previously observed in sepsis, where it was implicated in the *RUNX3–MAPK14*–miR-19b-1-5p–miR-5009-5p regulatory axis, highlighting its potential as a therapeutic target [42]. Based on this, we hypothesize that miR-5009-5p may possess tumour-suppressive properties in GB, and its upregulation by SHG-44 could contribute to anti-tumour effects. To explore its potential targets, we performed Sfold analysis, which showed that miR-5009-5p does not bind to *RUNX3*or *MAPK14* within the canonical 3′ UTR seed region (not shown). Thus, farther experimental validations are required to assess the role of this miRNA in GB tumorigenesis.

Our sequencing analysis identified miR-664b-3p as one of the top three upregulated miRNAs in response to SHG-44 treatment, suggesting a potential tumour suppressive role in GB. *In silico* expression analysis revealed that miR-664b-3p is significantly downregulated in GB patient samples when compared to controls, supporting the fact that its upregulation by SHG-44 may represent a favourable therapeutic effect (S1 Table). Previous studies have demonstrated that miR-664b-3p directly targets *Rheb*, a key regulator of the Rheb/mTOR/c-MYC signalling pathway, which promotes glycolysis and tumour growth in osteosarcoma (OS) cells [43]. This suggests that elevated levels of miR-664b-3p can suppress *Rheb* expression, thereby inhibiting downstream mTORC1 activity and reducing c-MYC-mediated metabolic reprogramming, a hallmark of cancer progression. To investigate this interaction, we performed Sfold binding site prediction analysis, which revealed that miR-664b-3p has three predicted binding sites in the 3′ UTR of *Rheb*. The most prominent was a 7mer-m8 site with a hybridization energy of –14.500 kcal/mol, indicating a strong and potentially functional interaction (S4 Fig). Further pathway analysis through KEGG indicated that miR-664b-3p is involved in the Ras signalling pathway, general pathways in cancer, and proteoglycans in cancer (S2 Table). Gene ontology analysis showed that the miRNA is primarily associated with protein binding (S3 Table). To date, the role of miR-664b-3p in GB has not been described, and its function in high-grade brain tumours remains largely unexplored. However, its upregulation in SHG-44-treated GB cells, combined with its predicted ability to suppress key oncogenic signalling pathways, suggests that miR-664b-3p may exert tumour suppressive effects, contributing to the anti-cancer activity of SHG-44.

Our findings highlighted the potential of SHG-44 to broadly influence cellular functions through its effects on miRNA expression. The altered expression of these miRNAs also suggested a potential impact on various biological processes such as axon guidance, which is crucial for neuronal connectivity and communication, and protein degradation, which is essential for maintaining cellular homeostasis. Additionally, cell adhesion pathways, critical for cell-cell interactions and tissue integrity were also affected. At the molecular level, the deregulated miRNAs were seen to interact with nucleic acids, zinc ions, sequence-specific DNA binding, and protein domain-specific binding. These interactions highlighted the various effects of SHG-44 at the cellular level through miRNAs expression modulation in GBs.

Recent evidence has demonstrated that trans chalcones possess the ability to cross the blood brain barrier (BBB) in a safe manner and to inhibit *VEGFR-2* in GB *in vivo* [44]. We performed further *in silico* analysis, which predicted that SHG-44 could pass freely through the BBB, addressing cytotoxicity-related challenges in GB treatment. Additionally, SHG-44 metabolites possessed low oral toxicity, which could improve patient management during therapy. Moreover, SHG-44 was not associated with carcinogenicity and none of the SHG-44 metabolites were predicted to be carcinogenic in the Ames test. Toxicological assessments of different chalcones have revealed that they possess very low genotoxicity [45]. Our *in-silico* results also depicted that SHG-44 was not genotoxic, suggesting its safer clinical application in GB therapy.

## Conclusion

In summary, the new chalcone compound SHG-44 demonstrated anti-cancer properties via inhibiting cell viability, proliferation and migration within GB cell models. The potential of the compound to modify the levels of GB-associated oncomiRs and tumour suppressor miRNAs was also observed. These preliminary *in vitro* results encourage further investigation regarding the effects of SHG-44 on miRNA regulation within GB cell models and its potential therapeutic application in the management of GB.

## Supporting information

S1 Fig Reduction in cell viability of HEK293 cells post exposure to SHG-44. **(A)** HEK293 cells exhibited a considerably higher IC₅₀ compared to GB cells, with IC₅₀ values of 179.27ΜM at 24h and **(B)** 189.04 ΜM at 48h. The data represent mean values and standard deviation, n = 3. **Legends:** ns: non-significant, *p < 0.05, **p < 0.01, ***p < 0.001, ****p < 0.0001.
(TIF)

S2 Fig Decreased clonogenic capacity of U87MG and U251MG GB cells following treatment with SHG-44 and cis-platin at equal concentrations. **(A)** A significant reduction in colony-forming ability was observed in U87MG cells treated with 100ΜM SHG-44 compared to 100ΜM cis-platin and the untreated control. **(B-C)** Microscopic imaging confirmed the inhibition of colony formation in U87MG cells following SHG-44 treatment, with no colonies evident. **(D)** Similarly, U251MG cells showed significantly decreased clonogenic potential upon 100ΜM SHG-44 treatment when compared to 100ΜM cis-platin and the untreated control. **(E-F)** Microscopic imaging confirmed the inhibition of colony formation in U251MG cells following SHG-44 treatment, with no colonies evident. Microscopic images were captured at ×40 magnification. Data represent mean values ± standard deviation, n = 3. **Legends:** ns: non-significant, *p < 0.05, **p < 0.01, ***p < 0.001, ****p < 0.0001.
(TIF)

S3 Fig Inhibition of cell migration in U87MG and U251MG GB cells following SHG-44 and cis-platin treatment at equal concentrations. **(A)** Quantification of wound closure in U87MG cells treated with DMSO (control), 100ΜM cis-platin, and 100ΜM SHG-44 at 0 h, 24 h, and 48h. There was no significant difference between cis-platin and SHG-44. **(B)** Quantification of wound closure in U251MG cells under the same treatment conditions and time points. There was no significant difference between cis-platin and SHG-44. **(C)** Representative scratch images from U87MG cells at 0 h, 24 h, and 48h following treatment with DMSO, cis-platin, or SHG-44. **(D)** Representative scratch images from U251MG cells at the same time points and conditions. Both SHG-44 and cis-platin significantly inhibited cell migration compared to the DMSO control, with no significant difference observed between the two treatments. Microscopic images of wound areas were taken at ×40 magnification. Data represent mean values ± standard deviation, n = 3. **Legends:** ns: non-significant, *p < 0.05, **p < 0.01, ***p < 0.001, ****p < 0.0001.
(TIF)

S4 Fig Predicted binding interactions between SHG-44-regulated miRNAs and target genes using Sfold analysis. **(A)** Sfold binding site prediction analysis identified a single binding site for miR-144-3p within the 3′ UTR of *c-Met*, classified as a 7mer-A1 site with a hybridization energy of –15.200 kcal/mol. **(B)** A similar 7mer-A1 binding site for miR-144-3p was predicted within the 3′ UTR of *TOP2A*, with a hybridization energy of –14.500 kcal/mol. **(C)** Sfold analysis revealed three predicted binding sites for miR-664b-3p in the 3′ UTR of *Rheb*, with the most prominent being a 7mer-m8 site and a hybridization energy of –14.500 kcal/mol.
(TIF)

S1 Table Differential expression of selected miRNAs across GB and other brain tumour datasets. Expression profiles of selected miRNAs were analysed across multiple publicly available datasets retrieved from the dbDEMC database. The table lists the miRNA name, dataset source ID, tumour type (GB: Glioblastoma, PA: Pilocytic Astrocytoma, LMD: Leptomeningeal Disease), number of cases analysed, expression status (upregulated or downregulated), and the corresponding log fold change (logFC) value compared to control samples.
(DOCX)

S2 Table KEGG pathway enrichment analysis of SHG-44-regulated miRNAs. Summary of the top three KEGG pathways significantly enriched for target genes of SHG-44-regulated miRNAs. The table includes the pathway name, number of target genes involved, associated miRNA, P-value, and false discovery rate (FDR).
(DOCX)

S3 Table Gene ontology enrichment analysis of SHG-44-regulated miRNAs. Summary of the top three enriched GO terms associated with the predicted target genes of SHG-44-regulated miRNAs. The table lists the GO term name, number of target genes involved, corresponding miRNA, P-value, and false discovery rate (FDR).
(DOCX)

S4 Table Apoptosis-related target genes of miR-122b-5p identified across KEGG signalling pathways. This table summarizes the 24 apoptosis-related target genes of miR-122b-5p identified through KEGG pathway analysis. The genes are categorized based on their association with four key pathways: the apoptosis pathway, PI3K-Akt signalling, p53 signalling, and the autophagy pathway. Each gene is classified as pro-apoptotic (20) or anti-apoptotic (4) based on its established role in apoptosis regulation.
(DOCX)

S1 File Raw data. All raw data required to replicate our study’s results, including values behind reported means, standard deviations, and data used to construct graphs, are included as a Supplementary Information file.
(XLSX)

S2 File Apoptosis target genes by pathway. List of 24 apoptosis-related target genes of miR-122b-5p, categorized by KEGG pathways (apoptosis, PI3K-Akt, p53 signaling, autophagy).
(XLSX)

## Abbreviations

SHG-44: *(E)*-1-(4-chlorophenyl)-3-(p-tolyl)prop-2-en-1-one
GB: Glioblastoma
miRNA: microRNA
CNS: Central nervous system
ESI: Electrospray ionization
WHO: Would Health Organization
TMZ: Temozolomide
ABCG2: ATP binding cassette subfamily G
NF-κB: Activated nuclear B cell growth
VEGF: Vascular endothelial growth factor
EGFR: Tyrosine kinase receptor
MET: Mesenchymal-epithelial transition factor
XN: Xanthohumol
RNA: Ribonucleic acid
DNA: Deoxyribonucleic acid
TLC: Thin layer chromatography
UV: Ultraviolet
IR: Infrared
HRMS: High resolution mass spectra
MTT: 3-(4,5-dimethylthiazol-2-yl)-2,5-diphenyltetrazolium bromide
DMSO: Dimethyl sulfoxide
PBS: Phosphate-buffered saline
PCR: Polymerase chain reaction
tRNA: Transfer RNA
rRNA: Ribosomal RNA
NCBI: National center for biotechnology information
KEGG: Kyoto encyclopaedia of genes and genomes
GO: Gene ontology
RT-qPCR: - Real time-quantitative polymerase chain reaction
SMILES: Simplified molecular-input line-entry system
NMR: Nuclear magnetic resonance
sRNA-seq: small RNA-sequencing
ROS: reactive oxygen species

## References

[1] Louis DN, Perry A, Wesseling P. The 2021 WHO Classification of Tumors of the Central Nervous System: A Summary. Neuro-oncology. 2021;23:1231–51. 34185076 10.1093/neuonc/noab106 PMC8328013

[2] Mohammed S, Dinesan M, Ajayakumar T. Survival and quality of life analysis in glioblastoma multiforme with adjuvant chemoradiotherapy: a retrospective study. Rep Pract Oncol Radiother. 2022;27(6):1026–36. doi: 10.5603/RPOR.a2022.0113 36632307 PMC9826661

[3] Fountain DM, Bryant A, Barone DG, Waqar M, Hart MG, Bulbeck H, et al. Intraoperative imaging technology to maximise extent of resection for glioma: a network meta-analysis. Cochrane Database Syst Rev. 2021;1(1):CD013630. doi: 10.1002/14651858.CD013630.pub2 33428222 PMC8094975

[4] Rong L, Li N, Zhang Z. Emerging therapies for glioblastoma: current state and future directions. J Exp Clin Cancer Res. 2022;41(1):142. doi: 10.1186/s13046-022-02349-7 35428347 PMC9013078

[5] Kao T-J, Lin C-L, Yang W-B, Li H-Y, Hsu T-I. Dysregulated lipid metabolism in TMZ-resistant glioblastoma: pathways, proteins, metabolites and therapeutic opportunities. Lipids Health Dis. 2023;22(1):114. doi: 10.1186/s12944-023-01881-5 37537607 PMC10398973

[6] Olivier C, Oliver L, Lalier L, Vallette FM. Drug resistance in glioblastoma: the two faces of oxidative stress. Front Mol Biosci. 2021;7:620677. 33585565 10.3389/fmolb.2020.620677 PMC7873048

[7] Salehi B, Quispe C, Chamkhi I, El Omari N, Balahbib A, Sharifi-Rad J, et al. Pharmacological Properties of Chalcones: A Review of Preclinical Including Molecular Mechanisms and Clinical Evidence. Front Pharmacol. 2021;11:592654. doi: 10.3389/fphar.2020.592654 33536909 PMC7849684

[8] Constantinescu T, Mihis AG. Two Important Anticancer Mechanisms of Natural and Synthetic Chalcones. Int J Mol Sci. 2022;23(19):11595. doi: 10.3390/ijms231911595 36232899 PMC9570335

[9] Ma Y, Cui Q, Zhu W, Wang M, Zhai L, Hu W, et al. A Novel Tetramethylpyrazine Chalcone Hybrid- HCTMPPK, as a Potential Anti-Lung Cancer Agent by Downregulating MELK. Drug Des Devel Ther. 2024;18:1531–46. doi: 10.2147/DDDT.S449139 38737331 PMC11088378

[10] Mendanha D, Vieira de Castro J, Moreira J, Costa BM, Cidade H, Pinto M, et al. A New Chalcone Derivative with Promising Antiproliferative and Anti-Invasion Activities in Glioblastoma Cells. Molecules. 2021;26(11):3383. doi: 10.3390/molecules26113383 34205043 PMC8199914

[11] Chen P-H, Chang C-K, Shih C-M, Cheng C-H, Lin C-W, Lee C-C, et al. The miR-204-3p-targeted IGFBP2 pathway is involved in xanthohumol-induced glioma cell apoptotic death. Neuropharmacology. 2016;110(Pt A):362–75. doi: 10.1016/j.neuropharm.2016.07.038 27487563

[12] Beylerli O, Gareev I, Sufianov A, Ilyasova T, Zhang F. The role of microRNA in the pathogenesis of glial brain tumors. Noncoding RNA Res. 2022;7(2):71–6. doi: 10.1016/j.ncrna.2022.02.005 35330864 PMC8907600

[13] Mustafov D, Siddiqui SS, Klena L, Karteris E, Braoudaki M. SV2B/miR-34a/miR-128 axis as prognostic biomarker in glioblastoma multiforme. Sci Rep. 2024;14(1):6647. doi: 10.1038/s41598-024-55917-6 38503772 PMC10951322

[14] Sufianov A, Begliarzade S, Ilyasova T, Liang Y, Beylerli O. MicroRNAs as prognostic markers and therapeutic targets in gliomas. Noncoding RNA Res. 2022;7(3):171–7. doi: 10.1016/j.ncrna.2022.07.001 35846075 PMC9271693

[15] Mustafov D, Siddiqui SS, Kukol A, Lambrou GI, Shagufta, Ahmad I, et al. MicroRNA-Dependent Mechanisms Underlying the Function of a β-Amino Carbonyl Compound in Glioblastoma Cells. ACS Omega. 2024;9(29):31789–802. doi: 10.1021/acsomega.4c02991 39072119 PMC11270567

[16] Braoudaki M, Lambrou GI, Papadodima SA, Stefanaki K, Prodromou N, Kanavakis E. MicroRNA expression profiles in pediatric dysembryoplastic neuroepithelial tumors. Med Oncol. 2016;33(1):5. doi: 10.1007/s12032-015-0719-3 26698155

[17] Yang Z, Wu L, Wang A, Tang W, Zhao Y, Zhao H, et al. dbDEMC 2.0: updated database of differentially expressed miRNAs in human cancers. Nucleic Acids Res. 2017;45(D1):D812–8. doi: 10.1093/nar/gkw1079 27899556 PMC5210560

[18] Tastsoglou S, Skoufos G, Miliotis M, Karagkouni D, Koutsoukos I, Karavangeli A, et al. DIANA-miRPath v4.0: expanding target-based miRNA functional analysis in cell-type and tissue contexts. Nucleic Acids Res. 2023;51(W1):W154–9. doi: 10.1093/nar/gkad431 37260078 PMC10320185

[19] Wishart DS, Tian S, Allen D, Oler E, Peters H, Lui VW, et al. BioTransformer 3.0-a web server for accurately predicting metabolic transformation products. Nucleic Acids Res. 2022;50(W1):W115–23. doi: 10.1093/nar/gkac313 35536252 PMC9252798

[20] Yang H, Lou C, Sun L, Li J, Cai Y, Wang Z, et al. admetSAR 2.0: web-service for prediction and optimization of chemical ADMET properties. Bioinformatics. 2019;35(6):1067–9. doi: 10.1093/bioinformatics/bty707 30165565

[21] Firmino PP, Queiroz JE, Dias LD, Wenceslau PRS, de Souza LM, Iermak I, et al. Synthesis, Molecular Structure, Thermal and Spectroscopic Analysis of a Novel Bromochalcone Derivative with Larvicidal Activity. Crystals. 2022;12(4):440. doi: 10.3390/cryst12040440

[22] Mphahlele MJ. Synthesis, Structural and Biological Properties of the Ring-A Sulfonamido Substituted Chalcones: A Review. Molecules. 2021;26(19):5923. doi: 10.3390/molecules26195923 34641467 PMC8512312

[23] Li W, Xu F, Shuai W, Sun H, Yao H, Ma C, et al. Discovery of Novel Quinoline-Chalcone Derivatives as Potent Antitumor Agents with Microtubule Polymerization Inhibitory Activity. J Med Chem. 2019;62(2):993–1013. doi: 10.1021/acs.jmedchem.8b01755 30525584

[24] Ouyang Y, Li J, Chen X, Fu X, Sun S, Wu Q. Chalcone Derivatives: Role in Anticancer Therapy. Biomolecules. 2021;11(6):894. doi: 10.3390/biom11060894 34208562 PMC8234180

[25] Bustos L, Echiburú-Chau C, Castro-Alvarez A, Bradshaw B, Simirgiotis MJ, Mellado M, et al. Cytotoxic Effects on Breast Cancer Cell Lines of Chalcones Derived from a Natural Precursor and Their Molecular Docking Analysis. Molecules. 2022;27(14):4387. doi: 10.3390/molecules27144387 35889260 PMC9318862

[26] Veliz EA, Kaplina A, Hettiarachchi SD, Yoham AL, Matta C, Safar S, et al. Chalcones as Anti-Glioblastoma Stem Cell Agent Alone or as Nanoparticle Formulation Using Carbon Dots as Nanocarrier. Pharmaceutics. 2022;14(7):1465. doi: 10.3390/pharmaceutics14071465 35890360 PMC9316063

[27] Bittencourt LFF, Oliveira KA de, Cardoso CB, Lopes FG, Dal-Cim T, Chiaradia-Delatorre LD, et al. Novel synthetic chalcones induces apoptosis in human glioblastoma cells. Chem Biol Interact. 2016;252:74–81. doi: 10.1016/j.cbi.2016.03.022 27012433

[28] Hba S, Ghaddar S, Wahnou H, Pinon A, El Kebbaj R, Pouget C, et al. Natural Chalcones and Derivatives in Colon Cancer: Pre-Clinical Challenges and the Promise of Chalcone-Based Nanoparticles. Pharmaceutics. 2023;15(12):2718. doi: 10.3390/pharmaceutics15122718 38140059 PMC10748144

[29] Jin J, Qiu S, Wang P. Cardamonin inhibits breast cancer growth by repressing HIF-1α dependent metabolic reprogramming. J Exp Clin Cancer Res. 2019;38(1):377. 31455352 10.1186/s13046-019-1351-4 PMC6712736

[30] Gil HN, Jung E, Koh D, Lim Y, Lee YH, Shin SY. A synthetic chalcone derivative, 2-hydroxy-3’,5,5’-trimethoxychalcone (DK-139), triggers reactive oxygen species-induced apoptosis independently of p53 in A549 lung cancer cells. Chem Biol Interact. 2019;298:72–9. doi: 10.1016/j.cbi.2018.11.003 30408460

[31] Martins T, Fonseca BM, Rebelo I. Antioxidant effects of chalcones during the inflammatory response: an overall review. Curr Med Chem. 2021;28:7658–713. 33992052 10.2174/0929867328666210511014949

[32] Merve Aydin E, Canıtez İS, Colombo E, Princiotto S, Passarella D, Dallavalle S, et al. Targeting Ovarian Cancer with Chalcone Derivatives: Cytotoxicity and Apoptosis Induction in HGSOC Cells. Molecules. 2023;28(23):7777. doi: 10.3390/molecules28237777 38067507 PMC10708092

[33] Yang Y, Wu H, Zou X, Chen Y, He R, Jin Y, et al. A novel synthetic chalcone derivative, 2,4,6-trimethoxy-4’-nitrochalcone (Ch-19), exerted anti-tumor effects through stimulating ROS accumulation and inducing apoptosis in esophageal cancer cells. Cell Stress Chaperones. 2022;27(6):645–57. doi: 10.1007/s12192-022-01302-z 36242757 PMC9672279

[34] Darwish MIM, Moustafa AM, Youssef AM. Novel tetrahydro-[1,2,4]triazolo[3,4a]isoquinoline chalcones suppress breast carcinoma through cell cycle arrests and apoptosis. Molecules. 2023;28(8):3338. 37110575 10.3390/molecules28083338 PMC10144155

[35] Wu Y, Chang J, Ge J. Isobavachalcone’s alleviation of pyroptosis contributes to enhanced apoptosis in glioblastoma: possible involvement of NLRP3. Molecular Neurobiology. 2022;59(11):6934–55. 36053436 10.1007/s12035-022-03010-2

[36] de Souza PS, Bibá GCC, Melo ED do N, Muzitano MF. Chalcones against the hallmarks of cancer: a mini-review. Nat Prod Res. 2022;36(18):4809–26. doi: 10.1080/14786419.2021.2000980 34865580

[37] Li H, Lei B, Xiang W, Wang H, Feng W, Liu Y, et al. Differences in Protein Expression between the U251 and U87 Cell Lines. Turk Neurosurg. 2017;27(6):894–903. doi: 10.5137/1019-5149.JTN.17746-16.1 27651343

[38] Komoto TT, Nishimura FG, Evangelista AF, de Freitas AJA, da Silva G, Silva WA, et al. Exploring the Therapeutic Potential of trans-Chalcone: Modulation of MicroRNAs Linked to Breast Cancer Progression in MCF-7 Cells. Int J Mol Sci. 2023;24(13):10785. doi: 10.3390/ijms241310785 37445965 PMC10341840

[39] Evers L, Schäfer A, Pini R, Zhao K, Stei S, Nimsky C, et al. Identification of Dysregulated microRNAs in Glioblastoma Stem-like Cells. Brain Sci. 2023;13(2):350. doi: 10.3390/brainsci13020350 36831894 PMC9953941

[40] Lan F, Yu H, Hu M, Xia T, Yue X. miR-144-3p exerts anti-tumor effects in glioblastoma by targeting c-Met. J Neurochem. 2015;135(2):274–86. 26250785 10.1111/jnc.13272

[41] Song J, Ma Q, Hu M, Qian D, Wang B, He N. The Inhibition of miR-144-3p on Cell Proliferation and Metastasis by Targeting TOP2A in HCMV-Positive Glioblastoma Cells. Molecules. 2018;23(12):3259. doi: 10.3390/molecules23123259 30544723 PMC6320803

[42] Luo X, Lu W, Zhao J, Hu J, Chen E, Fu S, et al. Identification of the TF-miRNA-mRNA co-regulatory networks involved in sepsis. Funct Integr Genomics. 2022;22(4):481–9. doi: 10.1007/s10142-022-00843-x 35322335

[43] Yu X, Duan W, Wu F, Yang D, Wang X, Wu J, et al. LncRNA-HOTAIRM1 promotes aerobic glycolysis and proliferation in osteosarcoma via the miR-664b-3p/Rheb/mTOR pathway. Cancer Sci. 2023;114(9):3537–52. doi: 10.1111/cas.15881 37316683 PMC10475784

[44] Senrung A, Tripathi T, Aggarwal N, Janjua D, Chhokar A, Yadav J, et al. Anti-angiogenic Potential of Trans-chalcone in an In Vivo Chick Chorioallantoic Membrane Model: An ATP Antagonist to VEGFR with Predicted Blood-brain Barrier Permeability. Cardiovasc Hematol Agents Med Chem. 2024;22(2):187–211. doi: 10.2174/0118715257250417231019102501 37936455

[45] Maronpot RR. Toxicological assessment of Ashitaba Chalcone. Food Chem Toxicol. 2015;77:111–9. 25576957 10.1016/j.fct.2014.12.021

