# Supplementary figures and images for: New chalcone compound exhibits microrna-mediated anticancer properties in glioblastoma
Source: PLoS One. 2025 Sep 26;20(9):e0330624. doi: 10.1371/journal.pone.0330624 (PMC12468763; doi:10.1371/journal.pone.0330624)

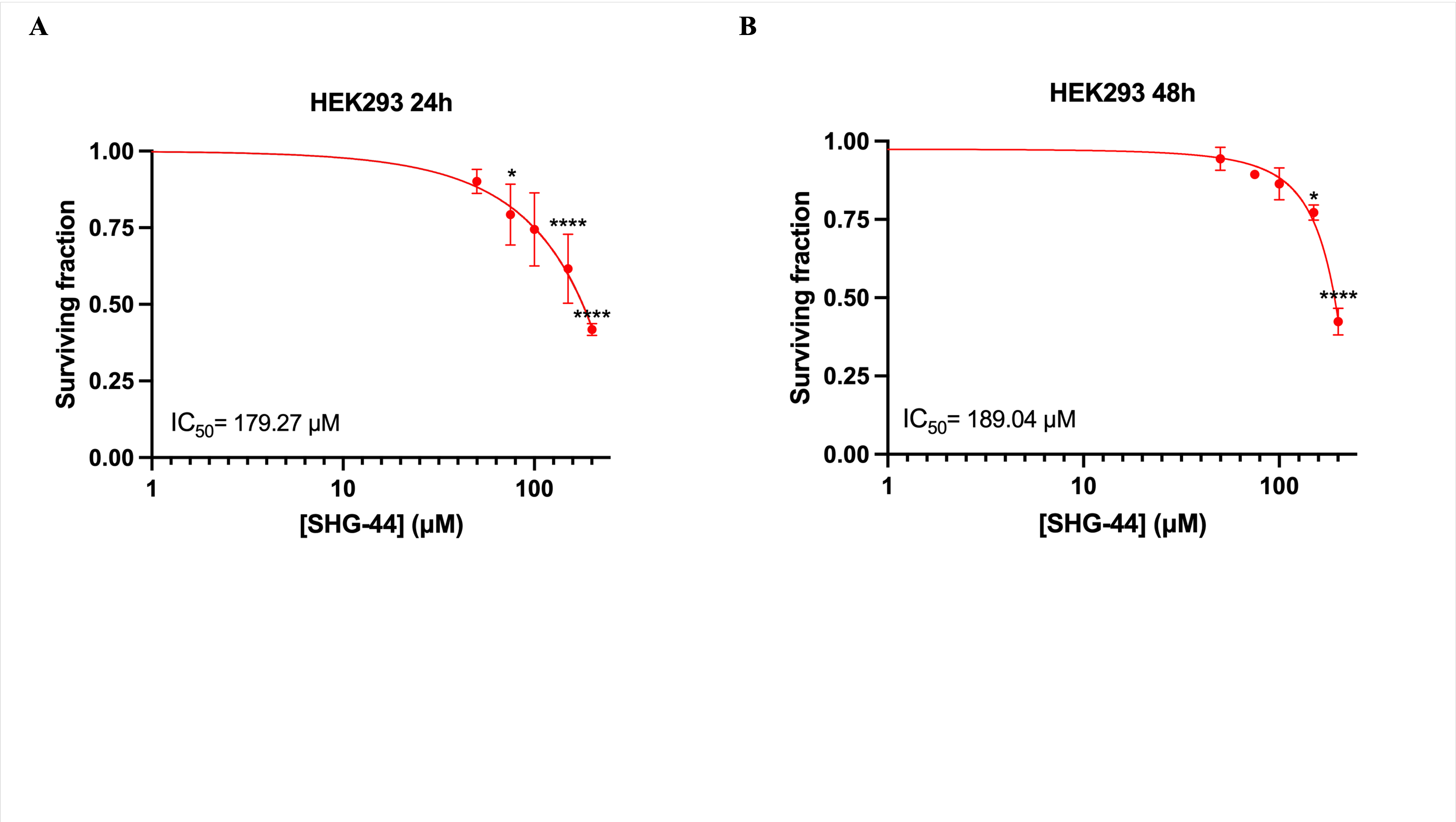

Supplement: S1 Fig — (A) HEK293 cells exhibited a considerably higher IC₅₀ compared to GB cells, with IC₅₀ values of 179.27ΜM at 24h and (B) 189.04 ΜM at 48h. The data represent mean values and standard deviation, n = 3. Legends: ns: non-significant, *p < 0.05, **p < 0.01, ***p < 0.001, ****p < 0.0001. (TIF) [file pone.0330624.s001.tif]

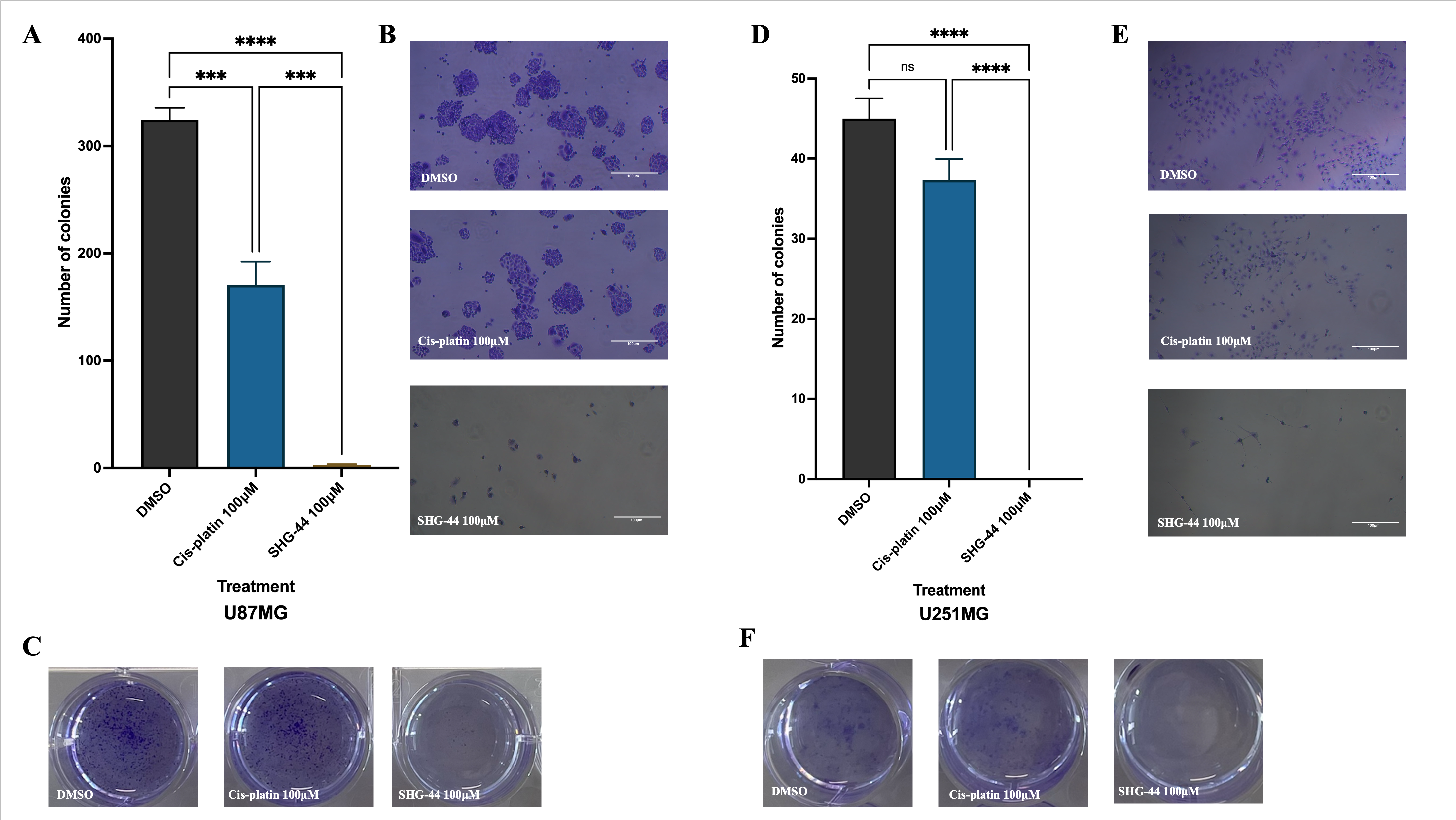

Supplement: S2 Fig — (A) A significant reduction in colony-forming ability was observed in U87MG cells treated with 100ΜM SHG-44 compared to 100ΜM cis-platin and the untreated control. (B-C) Microscopic imaging confirmed the inhibition of colony formation in U87MG cells following SHG-44 treatment, with no colonies evident. (D) Similarly, U251MG cells showed significantly decreased clonogenic potential upon 100ΜM SHG-44 treatment when compared to 100ΜM cis-platin and the untreated control. (E-F) Microscopic imaging confirmed the inhibition of colony formation in U251MG cells following SHG-44 treatment, with no colonies evident. Microscopic images were captured at ×40 magnification. Data represent mean values ± standard deviation, n = 3. Legends: ns: non-significant, *p < 0.05, **p < 0.01, ***p < 0.001, ****p < 0.0001. (TIF) [file pone.0330624.s002.tif]

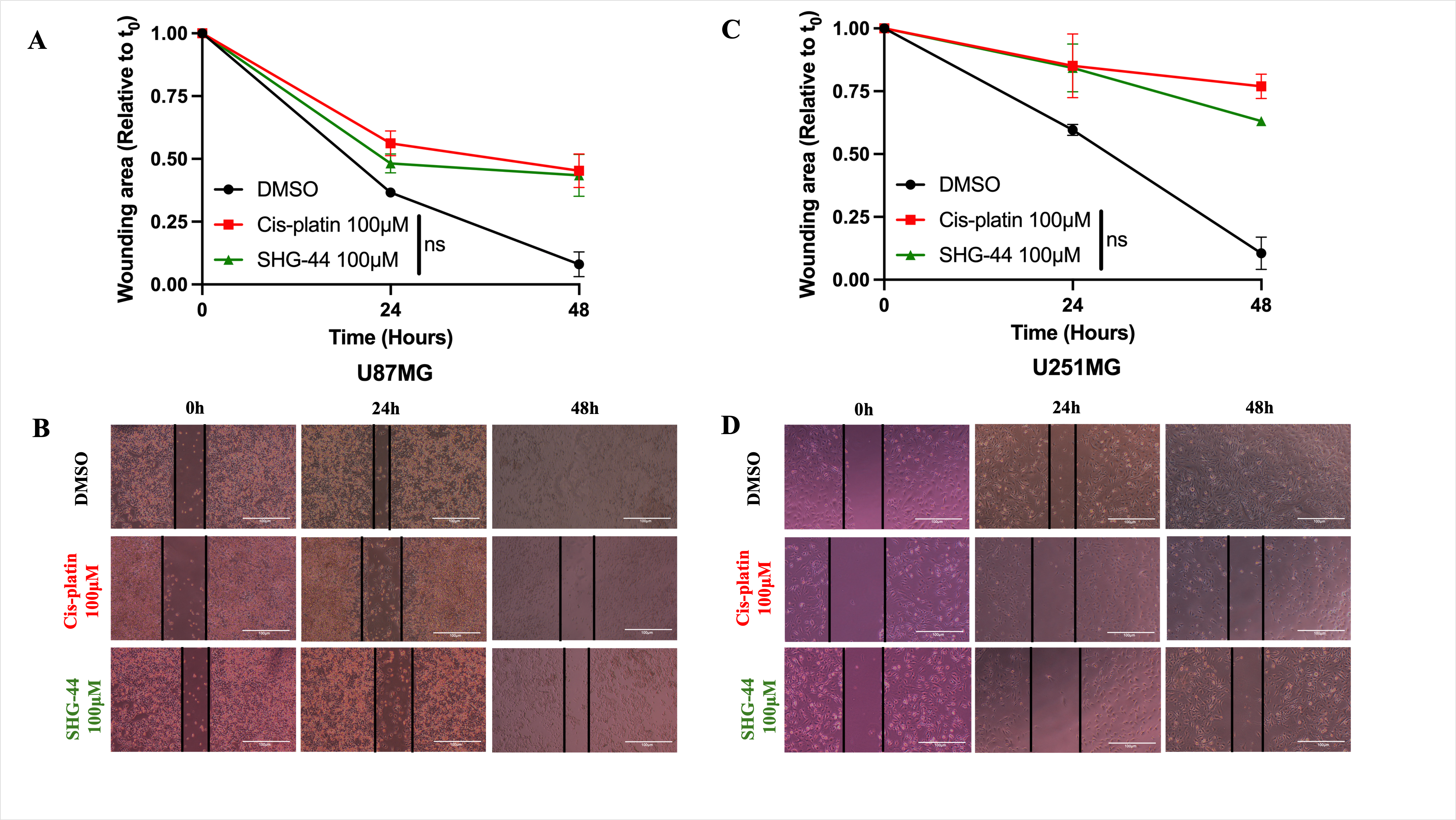

Supplement: S3 Fig — (A) Quantification of wound closure in U87MG cells treated with DMSO (control), 100ΜM cis-platin, and 100ΜM SHG-44 at 0 h, 24 h, and 48h. There was no significant difference between cis-platin and SHG-44. (B) Quantification of wound closure in U251MG cells under the same treatment conditions and time points. There was no significant difference between cis-platin and SHG-44. (C) Representative scratch images from U87MG cells at 0 h, 24 h, and 48h following treatment with DMSO, cis-platin, or SHG-44. (D) Representative scratch images from U251MG cells at the same time points and conditions. Both SHG-44 and cis-platin significantly inhibited cell migration compared to the DMSO control, with no significant difference observed between the two treatments. Microscopic images of wound areas were taken at ×40 magnification. Data represent mean values ± standard deviation, n = 3. Legends: ns: non-significant, *p < 0.05, **p < 0.01, ***p < 0.001, ****p < 0.0001. (TIF) [file pone.0330624.s003.tif]

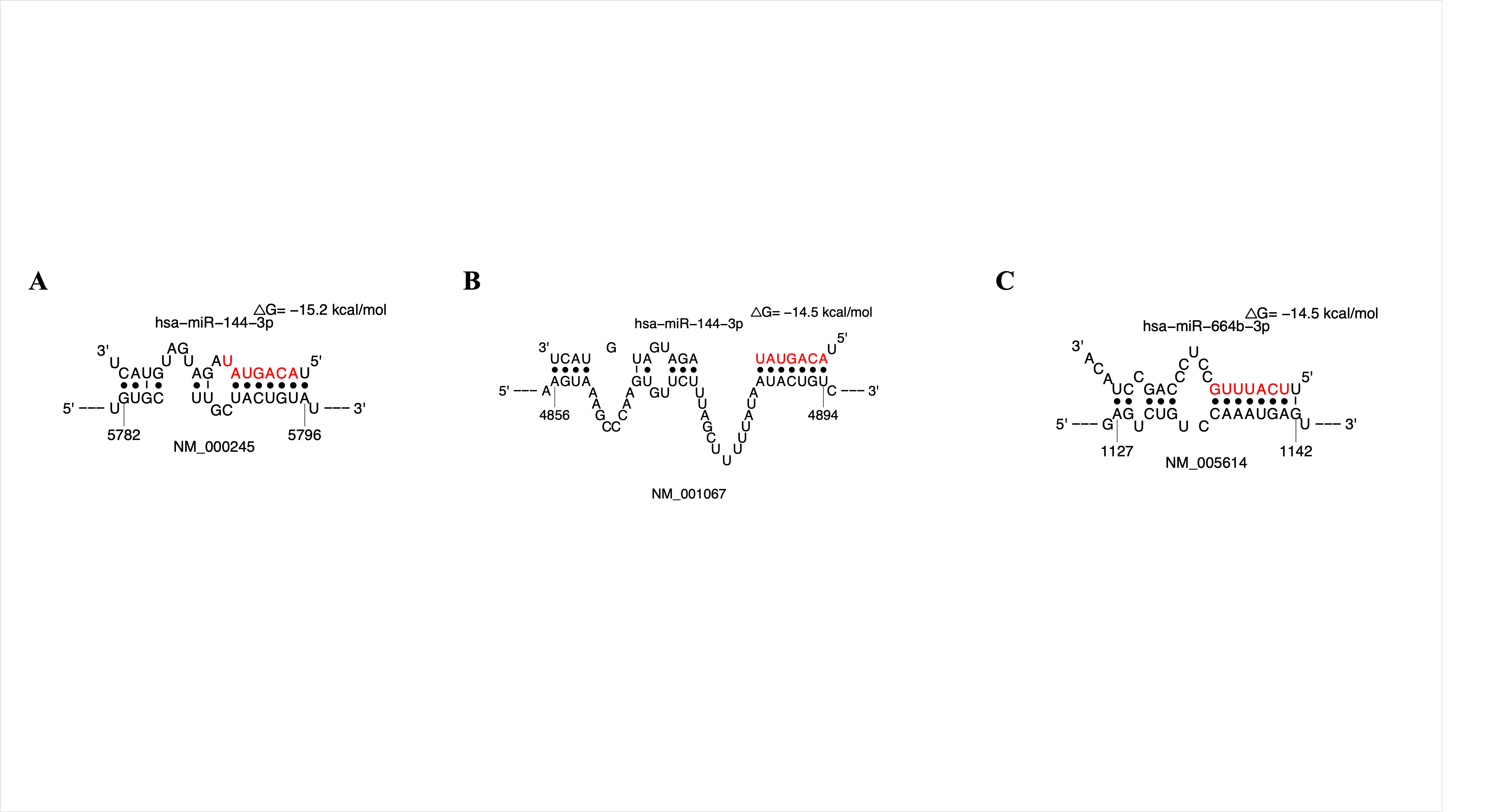

Supplement: S4 Fig — (A) Sfold binding site prediction analysis identified a single binding site for miR-144-3p within the 3′ UTR of c-Met, classified as a 7mer-A1 site with a hybridization energy of –15.200 kcal/mol. (B) A similar 7mer-A1 binding site for miR-144-3p was predicted within the 3′ UTR of TOP2A, with a hybridization energy of –14.500 kcal/mol. (C) Sfold analysis revealed three predicted binding sites for miR-664b-3p in the 3′ UTR of Rheb, with the most prominent being a 7mer-m8 site and a hybridization energy of –14.500 kcal/mol. (TIF) [file pone.0330624.s004.tiff]
