# Supplementary material for: New chalcone compound exhibits microrna-mediated anticancer properties in glioblastoma
Source: PLoS One. 2025 Sep 26;20(9):e0330624. doi: 10.1371/journal.pone.0330624 (PMC12468763; doi:10.1371/journal.pone.0330624)
Supplement: S1 Table — Expression profiles of selected miRNAs were analysed across multiple publicly available datasets retrieved from the dbDEMC database. The table lists the miRNA name, dataset source ID, tumour type (GB: Glioblastoma, PA: Pilocytic Astrocytoma, LMD: Leptomeningeal Disease), number of cases analysed, expression status (upregulated or downregulated), and the corresponding log fold change (logFC) value compared to control samples. (DOCX) [file pone.0330624.s005.docx]

| miRNA | Source ID | Tumour type | Cases | Expression status | logFC |
| --- | --- | --- | --- | --- | --- |
| miR-122b-5p | SRP262521 | GB | 35 | UP | 1.61 |
| miR-122b-5p | GSE138092 | LMD | 22 | UP | 1.69 |
| miR-223-3p | GSE135189 | PA | 16 | UP | 1.14 |
| miR-223-3p | GSE90603 | GB | 16 | UP | 2.02 |
| miR-223-3p | GSE113486 | GB | 40 | UP | 1.77 |
| miR-664b-3p | GSE113486 | GB | 40 | DOWN | -2.82 |
| miR-664b-3p | GSE139031 | GB | 170 | DOWN | -2.80 |
| miR-664b-3p | GSE112264 | GB | 50 | DOWN | -3.42 |
| miR-144-3p | GSE158284 | GB | 29 | UP | 4.21 |
| miR-144-3p | GSE135189 | PA | 16 | UP | 3.27 |
| miR-144-3p | GSE139031 | GB | 170 | UP | 1.03 |

**S1 Table. Differential expression of selected miRNAs across GB and other brain tumour datasets.** Expression profiles of selected miRNAs were analysed across multiple publicly available datasets retrieved from the dbDEMC database. The table lists the miRNA name, dataset source ID, tumour type (GB: Glioblastoma, PA: Pilocytic Astrocytoma, LMD: Leptomeningeal Disease), number of cases analysed, expression status (upregulated or downregulated), and the corresponding log fold change (logFC) value compared to control samples.
